# Precision nutrition for NAFLD: a mechanistic rationale for targeting the cytokine landscape

**DOI:** 10.3389/fcell.2026.1745316

**Published:** 2026-04-01

**Authors:** Amin Ullah, Shanshan Hu, Bairong Shen

**Affiliations:** Department of Endocrinology and Metabolism, Institutes for Systems Genetics, Frontiers Science Center for Disease-related Molecular Network, West China Hospital, Sichuan University, Chengdu, China

**Keywords:** curcumin, cytokines, NAFLD, omega-3, vitamins

## Abstract

Non-alcoholic fatty liver disease (NAFLD) represents a significant global health burden with limited approved pharmacotherapies. This therapeutic gap necessitates exploration of alternative strategies targeting the underlying inflammatory drivers of disease progression. NAFLD pathogenesis is characterized by chronic dysregulation of the cytokine milieu, promoting the transition from simple steatosis to steatohepatitis and fibrosis. This review critically evaluates mechanistic evidence for specific nutraceuticals—omega-3 fatty acids (EPA/DHA), vitamins D, E, and B, and curcumin—in modulating these immunometabolic pathways. Accumulating evidence indicates that these agents suppress pro-inflammatory mediators (e.g., IL-6, IL-1β) and enhance anti-inflammatory signals (e.g., IL-10) through inhibition of central regulators such as NF-κB and the NLRP3 inflammasome. However, their efficacy is context-dependent, with DHA demonstrating relatively consistent anti-fibrotic effects in preclinical models, curcumin exhibiting formulation- and bioavailability-dependent benefits, and EPA and vitamin D showing variable responses across studies and disease stages. Collectively, these findings highlight cytokine-targeted precision nutrition as a promising strategy for NAFLD management and underscore the need for robust clinical trials to establish personalized therapeutic protocols.

## Graphical Abstract

**Figure:**
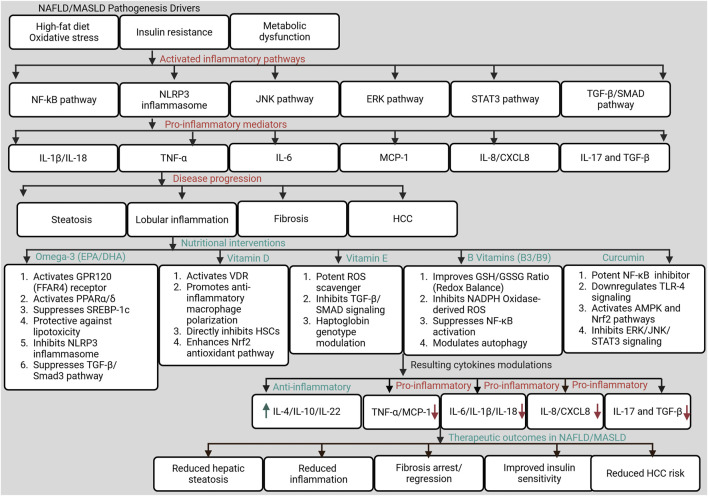

## 1 Introduction

The recent transition from the term non-alcoholic fatty liver disease (NAFLD) to metabolic dysfunction-associated steatotic liver disease (MASLD) reflects not merely a change in terminology but a revised diagnostic framework. NAFLD was historically defined by the presence of ≥5% hepatic steatosis in the absence of significant alcohol consumption or other secondary causes of liver fat accumulation (Maurice and Manousou, 2018). In contrast, MASLD requires evidence of hepatic steatosis together with at least one cardiometabolic risk factor—such as obesity, type 2 diabetes (T2D), hypertension, or dyslipidemia—thereby emphasizing metabolic dysfunction as a core component of disease classification (Girish and John, 2025). This redefinition emerged from international consensus discussions among major hepatology societies and represents a deliberate shift from an exclusion-based diagnosis toward a positive, etiology-driven framework that better reflects the underlying metabolic and inflammatory pathophysiology of the disease (Girish and John, 2025). The illness spectrum includes simple steatosis, non-alcoholic steatohepatitis (NASH), fibrosis, and hepatocellular carcinoma (HCC). Despite being frequently asymptomatic, NAFLD/MASLD is now recognized as the hepatic manifestation of metabolic syndrome and is closely linked to metabolic and cardiovascular comorbidities, including diabetes, insulin resistance (IR), obesity, hypertension, and dyslipidemia (Loomba et al., 2021). Its prevalence rose 50.4% worldwide between 1990 and 2019 (25.26%–38.00%), with the highest rates found in South Asia (33.83%), the Middle East/North Africa (36.53%), and Latin America (44.37%), while Western Europe had the lowest frequency (25.10%) (Younossi et al., 2023). Thus, NAFLD/MASLD is a multisystem metabolic disease with a substantial public health impact, underscoring the urgent need for mechanistic studies, early detection methods, and effective treatments.

In recent years, diet-based approaches and nutritional supplementation have gained extensive attention for managing NAFLD/MASLD. Nutraceuticals, which combine nutrition and pharmaceuticals, have emerged as particularly promising candidates (Cicero et al., 2018). Among the most studied are omega-3 (n-3) fatty acids, various vitamins, and the polyphenol curcumin (Vrentzos et al., 2025). The therapeutic potential of these agents is based on their multifaceted biological activities. n-3 polyunsaturated fatty acids (n-3 PUFAs), especially eicosapentaenoic acid (EPA) and docosahexaenoic acid (DHA), modulate inflammation and reduce triglycerides (Li et al., 2025). Furthermore, addressing deficiencies in vitamins B-complex, D, and E can counteract the oxidative stress and metabolic dysregulation driving NAFLD progression; consequently, vitamin supplementation presents a promising adjunct therapeutic avenue (Abe et al., 2021). Similarly, curcumin contributes potent antioxidant and anti-inflammatory properties (Jabczyk et al., 2021). Consequently, these nutraceuticals are now key focuses of late-phase clinical trials, evaluating their efficacy in reducing hepatic steatosis and inflammation through the modulation of lipid metabolism and immune responses (Hegazi et al., 2023).

Nutraceuticals may have an impact on cytokine networks, which are small, low-molecular-weight proteins that coordinate cell movement, proliferation, and interaction during tissue damage and inflammation for regulating immune responses (Ullah et al., 2022; 2023a; 2023b; 2024d; 2024c; 2024b; 2024a). Maintaining immunological homeostasis requires a balanced production of both pro- and anti-inflammatory cytokines (Marshall et al., 2018). Monocytes, macrophages, and T cells produce pro-inflammatory cytokines, including tumor necrosis factor-α (TNF-α), interleukin-6 (IL-6), IL-1, IL-8, and IL-17, which exacerbate inflammation, IR, and fibrosis, which accelerate the development of NASH. On the other hand, in NAFLD, anti-inflammatory cytokines such as IL-1 receptor antagonist (IL-1RA) and IL-10 reduce tissue damage and have beneficial effects (Ullah et al., 2023a; 2024c).

NAFLD/MASLD is increasingly recognized as a metabolically driven inflammatory disorder in which cytokine production arises from both hepatic and extra-hepatic sources. Within the liver, hepatocytes subjected to lipid overload and lipotoxic stress release damage-associated molecular patterns (DAMPs), which activate resident immune cells such as Kupffer cells, leading to the secretion of pro-inflammatory cytokines, including TNF-α, IL-1β, and IL-6. These cytokines further perpetuate inflammation and contribute to IR and hepatocellular injury (Katsarou et al., 2020). In addition to Kupffer cells, infiltrating monocyte-derived macrophages amplify the inflammatory milieu by secreting similar pro-inflammatory mediators and chemokines, contributing to disease progression from steatosis to steatohepatitis. Beyond the liver, dysfunctional adipose tissue in obesity secretes pro-inflammatory adipokines and cytokines (TNF-α, IL-6, MCP-1), driving systemic inflammation and indirectly exacerbating hepatic inflammation through increased free fatty acid flux and macrophage activation in the liver microenvironment (Katsarou et al., 2020; Ullah et al., 2024c).

NAFLD often coexists with other chronic inflammatory conditions, such as obesity, T2D, and metabolic syndrome, which are characterized by sustained low-grade inflammation. Elevated circulating cytokines in these conditions—particularly TNF-α and IL-6 — can exacerbate hepatic IR, promote lipid accumulation, and prime innate immune responses in the liver (Duan et al., 2022; Ullah et al., 2023a). These systemic cytokines activate key inflammatory pathways such as NF-κB and inflammasomes in hepatic cells, thereby accelerating progression from simple steatosis to non-alcoholic steatohepatitis (NASH), inflammation, and fibrogenesis. Dysregulation of interleukin signaling further contributes to immune cell recruitment and hepatic injury in the context of chronic inflammation beyond the liver itself (Duan et al., 2022).

Cytokines play distinct roles at different stages of NAFLD progression. In early steatosis, pro-inflammatory cytokines such as TNF-α and IL-6 are associated with hepatic IR and lipid accumulation. As the disease advances toward NASH, activation of inflammasome complexes, particularly NLRP3, leads to increased IL-1β production, hepatocyte injury, and neutrophil recruitment. In later stages, profibrogenic cytokines such as TGF-β become central drivers of HSC activation and extracellular matrix deposition, promoting fibrosis. These stage-dependent cytokine dynamics illustrate how inflammation evolves from a metabolic stress response toward a chronic immune-mediated pathology across the NAFLD spectrum (Katsarou et al., 2020; Guo et al., 2024).

Specific nutraceuticals target these immunometabolic pathways. For instance, n-3 fatty acids (EPA/DHA) reduce IL-6, TNF-α, IL-1β, and IL-8 while enhancing IL-10 and tumor growth factor-beta (TGF-β), in addition to generating specialized pro-resolving mediators that actively resolve inflammation (Jeyakumar and Vajreswari, 2021). Vitamin D (VD) reduces IR by activating hepatic VD receptors (VDR), lowering free fatty acids, and suppressing IL-6 and TNF-α, while enhancing insulin signaling (Sindhughosa et al., 2022). Curcumin has antioxidant properties, downregulates nuclear factor Kappa B (NF-κB) to inhibit pro-inflammatory cytokines, and activates AMP-activated protein kinase (AMPK) to promote fatty acid oxidation (Lukkunaprasit et al., 2023; Safari et al., 2023).

Taken together, these findings suggest that nutraceuticals play a pivotal role in modulating cytokine networks and may serve as effective adjuncts in the treatment of NAFLD. Therefore, this review aims to provide a comprehensive overview of the current evidence on how nutraceutical supplementation influences cytokine-driven immunological mechanisms in NAFLD/MASLD by integrating findings from both clinical and preclinical studies.

Several recent reviews have comprehensively summarized dietary patterns, nutraceutical interventions, and clinical outcomes in NAFLD/MASLD. However, most of these reports primarily emphasize clinical efficacy, dietary strategies, or general anti-inflammatory and antioxidant effects, without systematically dissecting the specific cytokine-driven signaling cascades that govern the transition from simple steatosis to steatohepatitis and fibrosis.

In contrast, the present review adopts a cytokine-centric mechanistic framework, organizing nutraceutical evidence according to their effects on defined inflammatory signaling axes, including NF-κB activation, NLRP3 inflammasome signaling, TGF-β/SMAD–mediated fibrogenesis, JAK/STAT pathways, macrophage polarization dynamics, and ferroptosis-associated inflammatory responses across hepatocytes, Kupffer cells, and HSCs.

Importantly, we critically evaluate context-dependent and sometimes paradoxical findings—such as the dissociation between steatosis improvement and cytokine modulation, divergent EPA *versus* DHA effects, and antioxidant *versus* direct immunomodulatory mechanisms of vitamin E (VE)—thereby addressing unresolved mechanistic controversies. By integrating molecular, cellular, and clinical evidence, this review extends beyond nutrient-focused summaries and provides a conceptual framework for cytokine-targeted precision nutrition strategies in NAFLD/MASLD. To our knowledge, no recent review has comprehensively structured nutraceutical evidence according to defined cytokine signaling axes while critically addressing context-dependent paradoxical responses.

## 2 Search criteria and data sources

The literature search was conducted using a structured strategy, as summarized in Table 1. Major databases were systematically searched using a comprehensive set of keywords related to fatty liver disease, specific nutraceuticals, and inflammatory cytokines to identify relevant peer-reviewed publications. The search was initially conducted between July and September 2025 for the original manuscript submission and was subsequently updated during manuscript revision in March 2026 to include additional relevant studies published up to November 2025.

**TABLE 1:** Structured literature review strategy.

Category	Description
Review type	Comprehensive (narrative) review with a systematically structured literature search
Initial search period	July–September 2025 (for the original manuscript submission)
Updated search	The literature search was updated during manuscript revision in March 2026 to include additional relevant studies published up to November 2025
Publication coverage	October 2001 – November 2025 (encompassing foundational studies through the most recent available evidence at the time of search)
Data sources	Web of Science, Google Scholar, PubMed/MEDLINE
Search terminology	Curcumin, Omega-3 Fatty Acids, Vitamin B, Vitamin D, Vitamin E, Fatty liver, Hepatic steatosis, Hepatic fibrosis, Non-alcoholic fatty liver disease (NAFLD), Non-alcoholic steatohepatitis (NASH), Metabolic dysfunction-associated steatotic liver disease (MASLD), Cytokines (IL-1β, IL-6, TNF-α, and others)
Inclusion criteria	The following criteria guided study selection: ➢ Peer-reviewed original research (human studies, animal models, *in vitro* studies) and scholarly review articles ➢ English language publications ➢ Studies directly investigating the relationship between the specified supplements and NAFLD/MASLD pathophysiology, with emphasis on inflammatory markers and hepatic pathology
Exclusion criteria	Studies were excluded if: ➢ They focused on liver diseases of other etiologies without clear relevance to NAFLD/MASLD ➢ They were conference abstracts, editorials, or opinion pieces without substantial data ➢ Full text was unavailable in English
Scope of evidence	The search included human studies (clinical trials), animal studies, and *in vitro* experiments. Review articles were also used to understand broader patterns in the research and to find additional original studies by checking their reference lists

## 3 Role of nutraceuticals in cytokine modulation in NAFLD

### 3.1 Omega-3 fatty acids and their effects on cytokines

**FIGURE 1:**
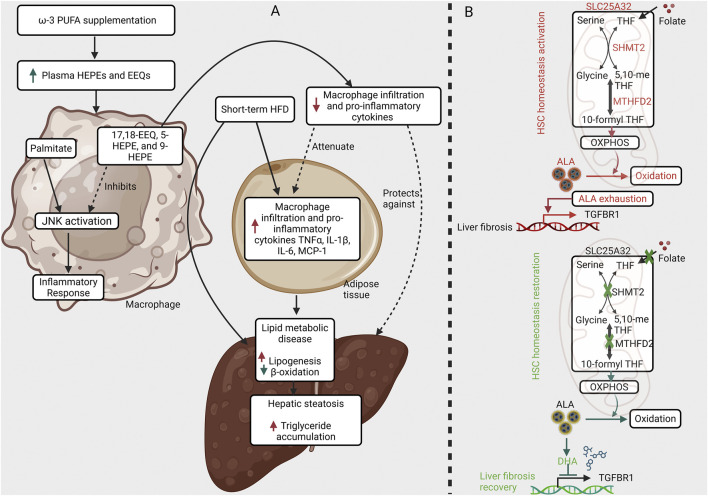
Metabolic pathways in liver disease ω-3 PUFA metabolites prevent steatosis while mitochondrial folate metabolism sustains fibrosis. **(A)** ω-3 PUFA metabolites attenuate adipose inflammation to protect against short-term high-fat diet (HFD)-induced hepatic steatosis. Short-term HFD triggers macrophage infiltration and pro-inflammatory cytokine release (TNFα, IL-1β, IL-6, CCL2) in adipose tissue, promoting hepatic steatosis *via* disrupted lipid metabolism. ω-3 PUFA supplementation generates metabolites (e.g., 17,18-EEQ, 5-HEPE, 9-HEPE) that inhibit palmitate-induced JNK activation in macrophages. This reduces adipose inflammation and its downstream effects on the liver, preventing steatosis (Solid arrows: induction; blunted red lines: inhibition). **(B)** Mitochondrial folate metabolism sustains profibrotic TGF-β1 signaling in hepatic stellate cells (HSCs). In HSCs, mitochondrial folate metabolism supports the persistence of TGF-β1 signaling by depleting α-linolenic acid (ALA) and enhancing TGF-β receptor 1 (TGF-βR1) expression. This establishes a feed-forward loop that amplifies the profibrotic activity of TGF-β1. Inhibition of mitochondrial folate metabolism interrupts this loop, weakening sustained TGF-β1 signaling and promoting the restoration of HSC quiescence and homeostasis.

In an animal model, a study has observed beneficial effects of PUFAs on NAFLD. In a study by Wang et al. (2017), a 4-day high-fat diet (HFD) was shown to induce lipid synthesis in C57BL/6 mice, an effect that was reversed by a short-term n-3 PUFA-enriched HFD (n-3HFD). Metabolomics analyses revealed that the short-term HFD reduced plasma levels of hydroxyeicosapentaenoic acids (HEPEs) and epoxyeicosatetraenoic acids (EEQs), which were subsequently increased following n-3PUFA supplementation. The n-3HFD also reduced macrophage infiltration in adipose tissue and lowered pro-inflammatory cytokines (IL-6, MCP-1, and TNF-α) in plasma. Using primary hepatocytes and peritoneal macrophages, the authors demonstrated that a mixture of 17,18-EEQ, 5-HEPE, and 9-HEPE attenuated the palmitate-induced activation of pro-inflammatory cytokines and the Jun N-terminal kinase (JNK) pathway. These findings identify these metabolites as active components and suggest their potential as a therapeutic strategy to prevent early NAFLD by suppressing adipose tissue macrophage infiltration and systemic inflammation *via* JNK signaling (Figure 1A) (Wang et al., 2017).

Mechanistically, EPA and DHA exert anti-inflammatory effects through specific signaling pathways. They activate the G-protein coupled receptor GPR120 and peroxisome proliferator-activated receptor gamma (PPARγ), leading to inhibition of NF-κB nuclear translocation and reduced transcription of pro-inflammatory cytokines such as TNF-α, IL-1β, and IL-6 in macrophages and hepatic immune cells. These effects are mediated, in part, through stabilization of IκB and suppression of NF-κB activation, thereby limiting pro-inflammatory gene expression (Calder, 2012). In addition, EPA and DHA serve as precursors of specialized pro-resolving mediators (SPMs) — including E-series and D-series resolvins, protectins, and maresins—which actively promote the resolution of inflammation by limiting neutrophil infiltration, enhancing macrophage efferocytosis, and facilitating the restoration of tissue homeostasis (Rasquel-Oliveira et al., 2023). Collectively, these pathways contribute to the inflammation-resolving and immunomodulatory properties of n-3 PUFAs in metabolic inflammatory conditions.

The anti-inflammatory benefits of n-3 PUFAs extend beyond early NAFLD to more advanced stages, such as NASH. In a rat model of NASH, n-3 fatty acid supplementation demonstrated significant anti-inflammatory properties by effectively modulating key cytokine pathways. Treatment with fish oil significantly reduced hepatic levels of the pro-inflammatory cytokines TNF-α and IL-6. Furthermore, it significantly lowered hepatic TGF-β1, a pleiotropic cytokine implicated in IR and fibrogenesis (Kabel et al., 2015). These findings indicate that the ameliorative effects of n-3 fatty acids on NASH are mediated, at least in part, through the suppression of a broad spectrum of inflammatory and profibrotic cytokines. Likewise, (Šmíd et al., 2023) demonstrated that dietary enrichment with n-3 PUFAs over a 6-week period in a high-fat methionine-and-choline-deficient (MCD) diet model resulted in significant decreases in hepatic expression of IL-1 and TNF-α, underscoring the potential of n-3 PUFAs to reduce inflammation associated with NAFLD.

Further supporting this trend, another study also reported positive outcomes across multiple health parameters. A diet rich in n-3 fatty acids improved all biochemical, inflammatory, and histological parameters tested. Linseed oil, which contains n-3 PUFAs, especially alpha-linolenic acid (ALA), has been shown to reduce body weight increase and visceral fat. These data reveal a direct relationship between the reduced expression of pro-inflammatory IL-6 and an increase in anti-inflammatory IL-10, as well as lower aspartate aminotransferase (AST) values, which are linked to an improvement in hepatic steatosis (Candido et al., 2020).

However, the anti-inflammatory effects of n-3 PUFAs are not consistently observed across all experimental models. In contrast, in this experimental murine model of NASH induced by an MCD diet, supplementation with a mixture of n-3 PUFAs (EPA and DHA) in olive oil significantly exacerbated hepatic inflammation compared to supplementation with olive oil alone (Provenzano et al., 2014). A marked increase in the intrahepatic mRNA expression of key pro-inflammatory mediators, including TNF-α and the chemokine MCP-1, evidenced this pro-inflammatory effect. It was associated with a significantly greater number of inflammatory lipogranulomas (Provenzano et al., 2014). The study demonstrates that in the context of this specific dietary model of NASH, n-3 PUFA administration promoted a more severe inflammatory phenotype, contrary to the anti-inflammatory effects often observed in other metabolic contexts.

In the studies above, most animal studies have demonstrated that n-3 PUFAs improve NAFLD/NASH by reducing liver fat and inflammation, primarily by lowering cytokines such as TNF-α and IL-6, and inhibiting JNK signaling. However, these effects are context-dependent, as evidenced by a study where n-3 PUFAs worsened inflammation in a specific MCD diet model, revealing that the benefits are not universal across all experimental conditions. A key limitation is the reliance on diverse animal models, particularly the use of the MCD diet, which does not fully represent human disease and may account for the discrepant results. Future work must define the specific conditions under which n-3 PUFAs are beneficial or detrimental to develop targeted and effective therapies for human NAFLD.

#### 3.1.1 EPA and cytokines

The n-3 PUFA (EPA) has been extensively studied for its beneficial effects on liver health. In a recent study, fish oil supplementation—rich in EPA—significantly reduced hepatic inflammation in low-density lipoprotein (LDL) receptor knockout mice fed an atherogenic HFD. This anti-inflammatory effect was evidenced by a marked downregulation of key pro-inflammatory cytokine and chemokine gene expression, including MCP-1 and IL-1α, with a strong trend toward reduced TNF-α expression (Harari et al., 2020). These molecular changes corresponded with improved histopathological scores for lobular inflammation and hepatocellular ballooning, indicating that EPA-rich fish oil attenuates cytokine-driven inflammatory pathways in the liver, contributing to its protective effects against NAFLD progression.

The anti-inflammatory mechanisms of n-3 PUFAs are complex and can be enhanced by synergistic combinations. n-3 long-chain polyunsaturated fatty acids (n-3 LCPUFAs) exhibit potent immunomodulatory actions, particularly when administered in combination with extra virgin olive oil (EVOO). In an HFD-induced steatosis model, this supplementation effectively suppressed elevations in the pro-inflammatory cytokines TNF-α and IL-6, an effect not achieved by either intervention alone. The anti-inflammatory potential of n-3 LCPUFAs is thought to involve peroxisome proliferator-activated receptor (PPAR)-α activation and the cross-inhibition of NF-κB signaling, alongside the generation of pro-resolving lipid mediators such as resolvins, protectins, and maresins, as well as Nrf2 activation, which counteracts oxidative stress and subsequent cytokine release (Valenzuela et al., 2016). Together, these mechanisms highlight the synergistic capacity of n-3 LCPUFAs and EVOO to attenuate systemic and hepatic inflammation, underscoring their therapeutic promise in mitigating diet-induced cytokine dysregulation and metabolic liver injury.

Further supporting the role of EPA, other marine-derived sources have shown similar potential to modulate inflammatory pathways. Dietary supplementation with marine-derived n-3 PUFAs has been shown to exert anti-inflammatory effects in metabolic disorders. In human TNFα-transgenic mice fed a HFD, herring roe and milt—rich sources of EPA—were associated with reduced plasma levels of several pro-inflammatory cytokines, including IL-1β, IL-2, IL-5, and granulocyte-macrophage colony-stimulating factor (CSF2). Although statistical significance was achieved only for IL-5 in the herring roe group, the overall cytokine profile suggested a trend toward diminished inflammatory signaling. These effects were accompanied by enhanced hepatic fatty acid catabolism and a reduction in the tissue n-6/n-3 PUFA ratio, underscoring the role of n-3 PUFAs in counteracting chronic TNFα-driven inflammation and metabolic dysfunction (Bjørndal et al., 2012).

Beyond general inflammation, EPA has also demonstrated a targeted ability to inhibit specific pro-tumorigenic pathways. In a murine model of obesity-related HCC, EPA supplementation demonstrated a protective effect by attenuating tumor development. Although it did not broadly ameliorate obesity-induced inflammation, EPA specifically targeted the pro-tumorigenic IL-6 pathway by suppressing the activation of its key downstream transcription factor, STAT3 (Inoue-Yamauchi et al., 2018). This inhibition of IL-6/STAT3 signaling represents a pivotal mechanism through which EPA exerts its anti-tumor activity, highlighting its potential clinical utility for the treatment of obesity-associated HCC.

However, the anti-inflammatory efficacy of EPA is not uniform across all contexts and can be influenced by specific molecular mechanisms. In db/db mice, supplementation with EPA-rich polar lipids from *Pyropia yezoensis* (SNL) significantly downregulated hepatic mRNA expression of the pro-inflammatory cytokine MCP-1, an effect further supported by a concurrent upregulation of the anti-inflammatory transcription factor PPARδ. However, the treatment’s inability to suppress TNF-α expression highlights a limitation in its anti-inflammatory efficacy, potentially restricting its effectiveness against the progression to advanced stages of NAFLD (Yanagita et al., 2020). In addition, (Du et al., 2013) uncovered a paradoxical role of EPA supplementation when mitochondrial β-oxidation is impaired. In mildronate-treated, carnitine-deficient mice, EPA markedly reduced systemic and tissue inflammation, shown by lower plasma TNF-α and IL-6, increased anti-inflammatory eicosanoids (Prostaglandin E_3_, Leukotriene B_5_), and downregulation of inflammatory genes such as MPEG1, COX2, and CD68 in liver and epididymal white adipose tissue. These effects were attributed to EPA’s incorporation into cellular lipids, which shifts eicosanoid profiles and may activate anti-inflammatory pathways, such as G-protein coupled receptor 120 (GPR120). Notably, these anti-inflammatory benefits were dissociated from hepatic lipid metabolism: rather than improving steatosis, EPA worsened it, increasing triglyceride accumulation and hepatotoxicity markers (Du et al., 2013). This dissociation carries important clinical implications: anti-inflammatory effects do not guarantee improved steatosis, and patients with mitochondrial dysfunction may not benefit from—or could be harmed by—EPA therapy. Future trials should therefore stratify participants by mitochondrial function to identify those most likely to respond safely. The findings indicate that while EPA exerts potent immunomodulatory effects, its therapeutic success in NAFLD requires functional mitochondrial β-oxidation, and it may be harmful in patients with mitochondrial impairment.

Despite these complexities, research also indicates that EPA can have direct antifibrotic effects, targeting later stages of NASH progression. Animal studies indicate that while EPA-ethyl ester (E) can accelerate the progression of existing hepatic fibrosis in NASH, it may also possess an antifibrotic effect. This beneficial impact is achieved by directly inhibiting the formation of reactive oxygen species (ROS), which is mediated through fatty acid oxidation and a TGF-β signaling pathway (Kajikawa et al., 2011). These mechanisms, which are separate from its ability to reduce triglyceride accumulation, could contribute to the therapeutic value of EPA-E for NASH patients. Supporting this, a few clinical pilot studies have shown that it may have a positive effect on reducing liver triglycerides and improving NAFLD. Using highly purified EPA-E, one of these studies found that serum ferritin and thioredoxin levels, which may indicate hepatic oxidative stress, as well as serum free fatty acids and plasma soluble TNF receptor 1 and 2 levels, were significantly reduced. In contrast, serum alanine aminotransferase (ALT) levels showed significant improvement. The levels of insulin, adiponectin, blood glucose, and body weight remained unchanged. Seven of the twenty-three patients who had post-treatment biopsies showed improvement in hepatic steatosis and fibrosis (Tanaka et al., 2008).

The above research demonstrates that EPA exerts anti-inflammatory, antifibrotic, and antitumorigenic effects in animal models of NAFLD/NASH by suppressing pro-inflammatory cytokines (e.g., TNF-α, IL-6, MCP-1), inhibiting NF-κB and STAT3 signaling, and activating PPAR pathways. Its efficacy can be enhanced through synergistic combinations, such as those with extra-virgin olive oil. However, these benefits are highly context-dependent. A critical paradox emerges where EPA reduces inflammation but exacerbates steatosis and hepatotoxicity under conditions of impaired mitochondrial β-oxidation, indicating that a functional metabolic state is essential for its safe therapeutic use. Promisingly, early clinical pilot studies have shown that EPA supplementation can improve biochemical markers, reduce hepatic triglycerides, and lead to histopathological improvement in steatosis and fibrosis for some patients (Tanaka et al., 2008; Kajikawa et al., 2011; Bjørndal et al., 2012; Du et al., 2013; Valenzuela et al., 2016; Inoue-Yamauchi et al., 2018; Harari et al., 2020).

#### 3.1.2 DHA and cytokines

DHA, an n-3 LCPUFA derived from the bioconversion of α-ALA, emerges as a crucial regulator of cytokine-driven fibrogenesis. This role is supported by extensive research into its anti-fibrotic and anti-inflammatory properties. Evidence from a recent study demonstrates that DHA effectively attenuates profibrotic cytokine signaling in hepatic stellate cells (HSCs) by inhibiting TGF-β1–induced Smad2/3 phosphorylation and downregulating the expression of classical fibrogenic markers, including α-SMA and collagen α1(I), in a dose-dependent fashion (Figure 1B) (Gao et al., 2023). Mechanistically, DHA reduced TGFBR1 mRNA expression, thereby disrupting the feedforward loop required for sustained TGF-β1 signaling and persistent HSC activation. Notably, the inhibitory effects of ALA on TGF-β1 signaling were contingent upon its conversion to DHA, underscoring DHA as the functional effector molecule (Gao et al., 2023). Taken together, these findings position DHA as a lipid-derived immunometabolic modulator that counteracts profibrotic cytokine and chemokine signaling, highlighting its therapeutic potential in promoting fibrosis resolution through the suppression of TGF-β1–mediated pathways.

Numerous animal studies have consistently demonstrated DHA’s potent capacity to suppress hepatic inflammation. DHA was shown to exert marked anti-inflammatory effects by modulating cytokine expression. In a recent study, DHA-enriched phosphatidylserine (DHA-PS) supplementation significantly attenuated hepatic inflammation in HFD-induced NAFLD mice by reducing pro-inflammatory cytokine levels. Specifically, DHA-PS treatment led to a marked decrease in IL-6, IL-1β, and TNF-α in liver tissue compared to the HFD control group, indicating potent anti-inflammatory effects that contribute to the amelioration of diet-induced metabolic inflammation (Zhou et al., 2021). In addition, in a murine model of NASH, DHA supplementation demonstrated potent anti-inflammatory effects by significantly suppressing hepatic pro-inflammatory cytokine expression, including TNF-α, and reducing macrophage infiltration, as indicated by F4/80. These immunomodulatory benefits were mediated specifically through GPR120/FFAR4 activation, as genetic ablation of the receptor abolished the cytokine-lowering and anti-inflammatory actions of DHA (Nakamoto et al., 2018). The findings underscore the role of GPR120 signaling in mediating the protective effects of DHA against cytokine-driven inflammation in NASH. Another study shows that DHA targets the TGFβ-Smad3 pathway to reduce hepatic fibrosis caused by a western diet (Lytle et al., 2015). In hepatic nuclei, DHA inhibits the buildup of phospho-Smad3, a crucial mediator of TGF-β-induced ECM synthesis, which is triggered by a western diet. Additionally, DHA suppresses the expression of several transcription factors associated with fibrosis, such as lysyl oxidases, collagen subtypes, matrix metalloproteases (MMPs), collagen structure, and tissue inhibitors of metalloproteases (TIMPs).

The consistency of DHA’s anti-inflammatory impact is highlighted by its ability to suppress a broad spectrum of pro-inflammatory cytokines across different models. In a murine model of HFD-induced NAFLD, DHA supplementation demonstrated potent anti-inflammatory properties by significantly downregulating hepatic mRNA expression of pro-inflammatory cytokines, including TNF-α, IL-1β, MCP-1, and IL-6. This suppression of cytokine gene expression, which was observed both *in vivo* and corroborated *in vitro* in human hepatoma cells, underscores a key mechanism through which DHA attenuates hepatic inflammation and contributes to the amelioration of NAFLD (Lin et al., 2014).

Beyond cytokine suppression, DHA also exerts its protective effects by targeting specific inflammatory signaling complexes. DHA exerts pronounced anti-inflammatory effects in the liver by targeting the NOD-like receptor protein 3 (NLRP3) inflammasome signaling cascade. Treatment with DHA has been shown to markedly reduce the hepatic mRNA expression of NLRP3 and pro-IL-1β, while simultaneously suppressing caspase-1 activation, thereby preventing the cleavage and secretion of mature IL-1β (Sui et al., 2016). Furthermore, DHA pretreatment effectively abolished lipopolysaccharide (LPS)-induced NLRP3 inflammasome activation both *in vitro* and *in vivo*. Mechanistic studies have attributed these effects to the inhibition of nuclear factor-kappa B (NF-κB) phosphorylation, a critical upstream regulator of inflammasome priming (Sui et al., 2016). Taken together, these findings highlight DHA as a potent modulator of inflammasome activity and cytokine release, underscoring its protective role against inflammatory mechanisms that drive the progression of NAFLD. In Table 2, we summarize the role of n-3 fatty acids, particularly DHA and EPA, in the modulation of cytokine pathways in NAFLD, highlighting evidence from both clinical and preclinical studies.

**TABLE 2:** The Role of Omega-3 Fatty Acids (Docosahexaenoic Acid [DHA], Eicosapentaenoic acid [EPA]) in NAFLD *via* Cytokine Modulation Based on Clinical and Preclinical Evidence. ↑ Increased expression/level, ↓ decreased expression/level, and ↔ expression/level unchanged.

Target cytokines	Study type/Trial number	Intervention detail/sample size	Internation duration	Outcome in NAFLD	Expression level of cytokines	Adverse effect/efficacy limitation	Ref
TNF-α, IL-1β, IL-6	Clinical/NCT00885313	DHA 250 mg/20 patients	18 months	After DHA treatment, improvements were observed in histopathological scores, hepatic progenitor cell activation, macrophage-driven inflammation, and hepatocyte G-protein coupled receptor 120/free fatty acid receptor 4 expression	↓	This study does not report any specific side effects or adverse events related to the 18-month DHA treatment	Nobili et al. (2014)
TNF-α	Clinical/ChiCTR-TRC-12002380	Fish oil (4 g), which contains EPA (728 mg) and DHA (516 mg/80 patients	3 months	After treatment, the levels of total cholesterol, triglycerides, fasting glucose, apolipoprotein B, alanine aminotransferase, and γ-glutamyl transpeptidase decreased	↓	No serious adverse events were reported. However, the corn oil (4 g) group showed a significant increase in serum creatinine, which was not observed in the fish oil group	Qin et al. (2015)
TNF-α, IL-1β, IL-6, IL-8, and IFN-γ	Clinical/NCT03528707	Omega-3 fatty acids 1%–5% + probiotic (250 mg)/48 patients	8 weeks	Probiotics with omega-3 significantly lowered serum gamma-glutamyl transpeptidase, triglycerides, and total cholesterol	↓	Both groups reported minor, self-resolving adverse events—mild abdominal pain, headache, and flatulence in the probiotic-omega group and nausea in two placebo patients—with comparable prevalence. No events affected treatment or led to withdrawal	Kobyliak et al. (2018)
TGF-β1	Preclinical	EPA/not mentioned	20 weeks	EPA suppresses lipogenesis *via* inhibition of SREBP-1c and enhances fatty acid β-oxidation *via* peroxisome proliferator-activated receptor α activation, and EPA reduces reactive oxygen species production	↓	The study did not report any adverse effects or side effects of EPA treatment; however, no significant change in AST and ALP was observed	Kajikawa et al. (2011)
TNF-α and IL-6	Preclinical	Omega-3 fatty acids (12 mL/kg)/not mentioned	2 weeks	After one to 2 weeks, omega-3 fatty acid treatment significantly reduced hepatic fat content, steatosis severity, the omega-6:3 ratio, and *de novo* lipogenesis, while improving antioxidative capacity and reducing inflammation	↓	This study does not report any specific side effects or adverse events related to the 2-week omega-3 fatty acid treatment	Marsman et al. (2011)
IL1RN, GDF15, TNF superfamily members, IL-17, IL-15, IL-1α IL-27, MCP-1, TNF-α and IL-6	Preclinical	DHA 40%/not mentioned	30 weeks	Treatment with DHA prevented fibrosis progression, reduced hepatic lipid content, and improved lipid profile	↓	No adverse effect was reported in this study during treatment with DHA. However, it did not reverse metabolic syndrome features (obesity, hyperglycemia) or fully resolve liver injury (ALT levels did not significantly improve)	Lytle et al. (2017)
IL-6 and TNF-α	Preclinical/NAFLD and HCC model	EPA 5%/not mentioned	9 months	EPA significantly reduced liver triglyceride and total cholesterol levels, improved liver histology, and decreased liver weight, as well as plasma aspartate transaminase/alanine transaminase levels	↔	No adverse effects were reported in this study. However, EPA did not reduce body weight gain induced by HFD and did not decrease the number of tumors, although it significantly reduced tumor size. No effect on apoptosis was observed, as TUNEL staining remained unchanged	Inoue-Yamauchi et al. (2018)
IL-6, TNF-α, MCP-1 and IL-1β	Preclinical	DHA 1%/not mentioned	4 weeks	DHA ameliorates NAFLD by reducing hepatic lipid accumulation, inflammation, and serum markers of liver injury. DHA primarily targets lipogenesis and inflammation	↓	No adverse effects were reported in this study	Lin et al. (2014)

In the above studies, DHA demonstrates potent anti-fibrotic and anti-inflammatory effects in NAFLD/NASH through multiple mechanisms. It directly inhibits TGF-β1/Smad signaling in HSCs, thereby reducing fibrosis and suppressing pro-inflammatory cytokines (TNF-α, IL-6, IL-1β) through the inhibition of the GPR120/NF-κB pathway and modulation of the NLRP3 inflammasome. Unlike EPA, DHA consistently shows beneficial effects across studies without exacerbating steatosis, positioning it as a promising therapeutic agent for targeting both inflammation and fibrosis in liver disease.

### 3.2 Vitamin D (VD) and its effects on cytokines

#### 3.2.1 VD and cytokines

Recent evidence suggests that VD may have immunomodulatory properties in NAFLD. In an HFD–induced NAFLD mouse model, supplementation with active VD markedly attenuated systemic inflammation, as reflected by reduced circulating levels of the pro-inflammatory cytokine TNF-α. Interestingly, the anti-inflammatory effect did not appear to result from a direct and broad suppression of cytokine activity. Instead, it was mechanistically attributed to the inhibition of the p53 signaling pathway, which in turn mitigated hepatocyte senescence and apoptosis (Liu et al., 2020). These findings suggest that VD alleviates NAFLD-associated inflammation primarily through the upstream regulation of cellular stress and senescence processes, rather than through direct modulation of the inflammatory cytokine network. Other studies suggest that VD may slow the proliferation of HSCs (reviewed by Kwok et al. (2013)). Together, these observations indicate that VD may affect liver fibrosis controlled by HSCs through multiple mechanisms.

Advancing from the foundational research in mouse models, subsequent studies have explored novel delivery methods to enhance VD’s efficacy. VD, particularly when delivered *via* a nanoemulsion, demonstrates significant therapeutic potential. Research by El-Sherbiny et al. shows that a VD nanoemulsion effectively combats inflammation and oxidative stress in HFD rats. Its superior efficacy over standard VD is attributed to enhanced activation of the Nrf2 antioxidant pathway, a greater reduction in the pro-inflammatory cytokine TNFα, and an increase in the anti-inflammatory cytokine IL-10. Furthermore, it improved liver health by reducing steatosis and fibrosis, boosting fat-burning (Cpt1a mRNA), and lowering lipid levels (El-Sherbiny et al., 2018).

Similarly, Trépo et al. (2013) found that a severe VD shortage was substantially linked to complications from portal hypertension and a poor prognosis. This work further confirmed the significance of VD in immunomodulation by demonstrating that VD treatment or supplementation can inhibit the expression of the pro-inflammatory cytokine TNF-α. Moreover, in HFD-induced NASH models, calcitriol (1,25-dihydroxyvitamin D_3_) treatment has been demonstrated to reduce inflammation. The treatment suppressed the expression of TNFR1 and NF-κB while lowering pro-inflammatory cytokines (TNF-α, IL-6, and MCP-1) in the liver, mesenteric adipose tissue, and circulating monocytes. These effects were mediated through restoration of VDR signaling, which inhibited TNF-α–TNFR1–NF-κB pathways, improved intestinal barrier integrity, lowered endotoxemia, and ameliorated hepatic steatosis (Su et al., 2018). Overall, VD rebalanced the cytokine milieu toward an anti-inflammatory state, highlighting its therapeutic potential in NASH.

**FIGURE 2:**
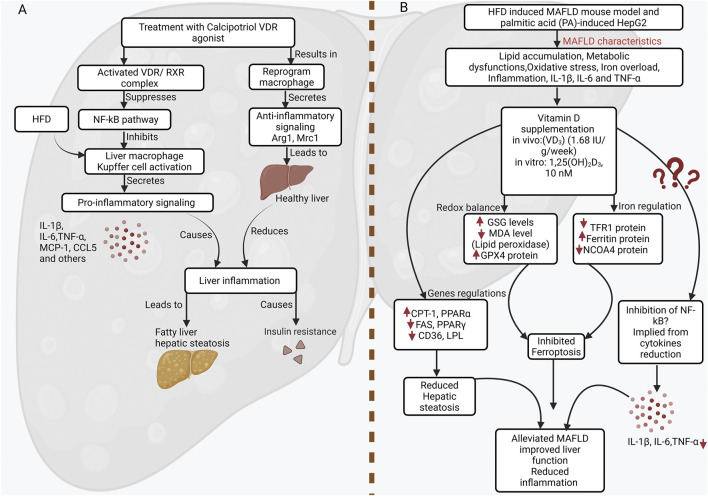
Vitamin D protects against MAFLD by reprogramming macrophages and regulating lipid metabolism, ferroptosis, and inflammation. **(A)** Mechanism of vitamin D receptor (VDR) agonist action in hepatic macrophages. A high-fat diet activates liver macrophages (Kupffer cells), promoting pro-inflammatory signaling through the NF-κB pathway. This leads to the secretion of cytokines (IL-1β, IL-6, TNF-α) and chemokines (MCP-1/CCL2, CCL5), which drive hepatic inflammation, steatosis, and insulin resistance (IR). Treatment with the VDR agonist calcipotriol promotes the formation of an activated VDR/retinoid X receptor (RXR) complex. This complex suppresses the NF-κB pathway, inhibiting macrophage activation. Consequently, the macrophage is reprogrammed to an anti-inflammatory state, secreting factors like Arg1 and Mrc1, which resolve inflammation, reduce steatosis, and restore insulin sensitivity to promote a healthy liver. **(B)** Vitamin D alleviates MAFLD by targeting lipid metabolism, ferroptosis, and inflammation. In HFD-fed mice and PA-treated HepG2 cells, Vitamin D supplementation improves lipid homeostasis (modulating CPT-1, PPARα, FAS, PPARγ, CD36, LPL), inhibits ferroptosis (restoring iron balance *via* TFR1, Ferritin, NCOA4, and reducing lipid peroxidation), and suppresses pro-inflammatory cytokines (TNF-α, IL-1β, IL-6). These coordinated actions reduce hepatic steatosis and improve liver function.

The immunomodulatory effects of VD extend to reshaping the entire hepatic immune environment. In C57BL/6 mice fed an HFD, supplementation with VD, alone or combined with *Lentinula edodes* extracts, significantly reduced body fat accumulation, liver fat content, serum triglycerides, and LDL-cholesterol, while increasing the high-density lipoprotein (HDL)/LDL ratio. These metabolic benefits were accompanied by marked immunomodulatory effects, including reduced pro-inflammatory cytokines (TNF-α, IL-1α, IL-1β), increased TGF-β1, and trends toward elevated IL-10, leading to higher IL-10/TNF-α and IL-4/TNF-α ratios. VD also enhanced the CD4/CD8 lymphocyte ratio, indicating improved adaptive immune balance. Together, these findings demonstrate that VD fosters an anti-inflammatory cytokine milieu that mitigates liver injury and protects against diet-induced NASH (Drori et al., 2017). Dong and colleagues have shown that in a diet-induced model of NASH, VDR activation reduces hepatic inflammation (Dong et al., 2020). In comparison to vehicle-treated controls, mice treated with the VDR agonist calcipotriol had improved IR, reduced steatosis, and lower hepatic inflammation. Additionally, it was demonstrated that calcipotriol mainly affects hepatic macrophages because the treatment’s impact was reversed when hepatic macrophages were depleted using clodronate liposomes (Figure 2A) (Dong et al., 2020).

The modulation of macrophage activity and polarization represents a central mechanism for VD’s immunomodulatory effects. Recent findings suggest that 1,25-dihydroxyvitamin D_3_ (1,25 VD3) supplementation exerts potent immunomodulatory effects in NASH. In a diet-induced NASH rat model, administration of 1,25 VD3 was shown to reshape the hepatic cytokine milieu by suppressing pro-inflammatory mediators such as TNF-α and IL-1β, while simultaneously enhancing anti-inflammatory cytokines, including IL-10 and IL-4. This immunological shift was mechanistically associated with inhibition of NF-κB signaling and activation of PPARγ, pathways well recognized for their opposing roles in inflammation and metabolic regulation (Liu et al., 2025). Moreover, these molecular changes were accompanied by a phenotypic switch in macrophage polarization, favoring the anti-inflammatory M2 subset over the pro-inflammatory M1 phenotype (Liu et al., 2025). Collectively, these findings highlight the capacity of 1,25 VD3 to attenuate NASH-related inflammation through coordinated modulation of cytokine signaling and immune cell plasticity, underscoring its therapeutic potential as an immunoregulatory agent in metabolic liver disease. Furthermore, in an experimental model of NASH induced in ovariectomized mice, supplementation with 1,25(OH)_2_D_3_ demonstrated modest reductions in hepatic mRNA expression of the pro-inflammatory cytokines TNF-α and IL-6; however, these effects did not reach statistical significance. Notably, the principal therapeutic benefits of VD supplementation appeared to derive from its capacity to attenuate fibrogenic signaling, as evidenced by significant downregulation of TGF-β and suppression of its downstream SMAD pathway (Ma et al., 2019). This suggests that the anti-fibrotic and anti-inflammatory actions of VD in NASH may be mediated predominantly through modulation of profibrotic signaling cascades rather than direct inhibition of classical inflammatory cytokines. In addition, treatment with 1,25-(OH)_2_D_3_ markedly reduced serum levels of TNF-α and decreased hepatic TNF-α immunoreactivity, demonstrating a potent systemic and local anti-inflammatory effect. This suppression of cytokine expression underscores VD’s role in mitigating the chronic inflammatory state that is central to NAFLD progression (Ibrahim et al., 2023). In a recent study, VD supplementation with 1,25(OH)_2_D_3_ significantly reduced the pro-inflammatory cytokine response by inhibiting M1 macrophage polarization. Treatment decreased the expression and secretion of key M1-associated cytokines, including TNF-α, IL-6, and IL-1β, in both hepatic macrophages *in vivo* and in palmitic acid-stimulated RAW264.7 macrophages *in vitro*. This suppression of cytokine production was mediated through the VDR-PPARγ pathway, highlighting a mechanism by which VD exerts its anti-inflammatory effects in NAFLD (Luo et al., 2024). Likewise, VD supplementation significantly attenuated the pro-inflammatory cytokine response in a HFD-induced NAFLD model. Treatment reduced both the protein and mRNA expression levels of key inflammatory mediators, including TGF-β1, IL-1β, and TNF-α, in liver tissue. This suppression of cytokine production was associated with a downstream reduction in oxidative stress, as indicated by decreased malondialdehyde levels (Chung et al., 2025). The findings demonstrate that VD exerts potent anti-inflammatory effects, contributing to the amelioration of steatohepatitis and hepatic fibrosis.

The protective role of VD is further evidenced by its impact on broader pathological mechanisms beyond pure inflammation. A recent study found that VD supplementation protects against metabolic-associated fatty liver disease (MAFLD) by reducing systemic inflammation and improving metabolic regulation. In HFD-induced mice, VD_3_ lowered pro-inflammatory cytokines (TNF-α, IL-6, IL-1β), thereby mitigating hepatic injury, IR, and fibrosis. Mechanistically, VD alleviated oxidative stress and inhibited ferroptosis through increased antioxidant capacity (↑GSH, ↓MDA), reduced iron overload, and regulation of ferroptosis-related proteins. These combined effects highlight VD as a multifaceted modulator of inflammation, oxidative stress, and cell death in MAFLD (Figure 2B) (Miao et al., 2025).

Direct effects on HSCs also support the anti-fibrotic properties of VD. The extracellular matrix secreted by HSCs serves as a scaffold for the formation of fibrotic tissue and cellular regeneration. It is possible for active 1,25(OH)2D to directly inhibit these cells’ capacity to produce type I collagen (Potter et al., 2013). Research on HSCs isolated from patients with morbid obesity and biopsy-confirmed NAFLD revealed an association between advanced liver fibrosis and the proteolytic fragmentation of the VDR (Beilfuss et al., 2015). The study further showed that *in vitro* administration of 1 μM vitamin D2 (VD2) inhibited the TGFβ-induced pro-fibrogenic activity of HSCs, an effect mediated through modulation of SMAD2 protein expression.

**FIGURE 3:**
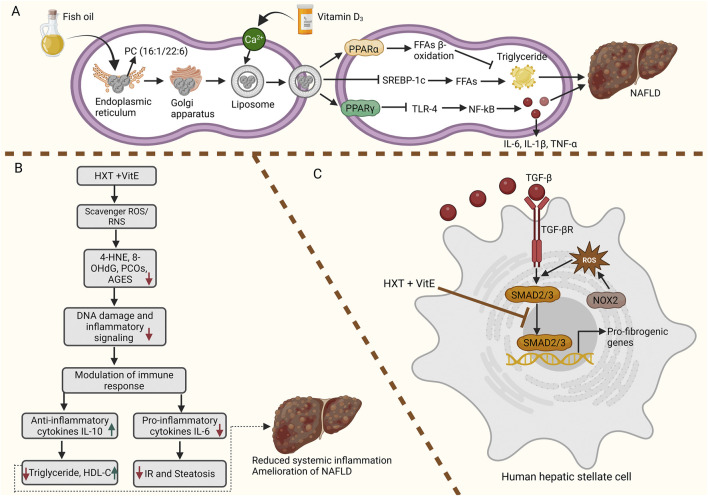
Therapeutic strategies in NAFLD phosphatidylcholine (PC) (16:1/22:6) improve lipid metabolism and inflammation while hydroxytyrosol (HXT) and vitamin E (VitE) synergistically reduce oxidative stress, modulate immune responses, and suppress fibrosis. **(A)** Potential mechanisms by which PC (16:1/22:6) reduces NAFLD. PC (16:1/22:6) may increase PPARγ activity, suppressing the TLR-4/NF-κB signaling cascade and lowering pro-inflammatory cytokines such as IL-1β and TNF-α. Additionally, it can lower both circulating and hepatic free fatty acid levels by inhibiting SREBP-1c–driven lipogenesis. Furthermore, PC (16:1/22:6) promotes PPARα-mediated fatty acid β-oxidation in the liver, which helps counteract lipid peroxidation and mitigates hepatic injury. **(B)** The combined antioxidant treatment of HXT and VitE ameliorates paediatric NAFLD by targeting the core pathways of the disease. The therapy directly reduces oxidative stress, evidenced by the significant decrease in the DNA damage marker 8-OHdG and the lipid peroxidation product 4-HNE. Following this decrease in oxidative damage, the systemic inflammatory response is modulated, resulting in a significant rise in the anti-inflammatory cytokine IL-10 and a drop in the pro-inflammatory cytokine IL-6. Ultimately, these molecular changes are directly correlated with key clinical improvements: the rise in IL-10 and fall in oxidative stress are linked to improved blood lipid profiles (lower triglycerides, higher HDL-cholesterol), while the reduction in both IL-6 and 8-OHdG is associated with the amelioration of liver steatosis and IR. Thus, HXT and VitE work not merely as simple antioxidants but as modulators of the immune response, breaking the cycle of oxidative stress and inflammation that drives NAFLD progression. **(C)** HXT and VitE synergistically disrupt TGF-β-driven fibrotic signaling. Combined treatment with HXT and VitE counteracts TGF-β-induced ROS accumulation by hindering the nuclear translocation and activity of SMAD2/3 transcription factors. This suppression reduces the expression of downstream target genes, including COL1A1, COL3A1, NOX2, and TGF-β itself, thereby interrupting the self-perpetuating cycle of fibrosis.

Translating these mechanistic insights into human applications, clinical trial has begun to validate the immunomodulatory role of VD supplementation. Recent evidence from a randomized controlled trial highlights the potential mechanistic link between combined fish oil and VD_3_ (FO + D) supplementation and lipid-mediated immune modulation. Notably, this intervention led to a significant increase in serum levels of the phospholipid phosphatidylcholine (PC) (16:1/22:6). It is noteworthy that a substantial and inverse correlation was observed between high PC (16:1/22:6) and circulating levels of TNF-α and IL-1β. These findings suggest that the anti-inflammatory properties attributed to FO + D may be mediated, at least in part, through the upregulation of specific lipid species such as PC (16:1/22:6), which in turn appears to modulate cytokine production and attenuate systemic inflammation (Figure 3A) (Fan et al., 2022). VD has been shown to influence inflammatory responses in NAFLD by modulating cytokine production and immune cell activity. Both clinical and experimental studies support its role in attenuating liver inflammation and fibrosis, as outlined in Table 3.

**TABLE 3:** The Role of vitamins (D, E, and B) in NAFLD *via* Cytokine Modulation Based on Clinical and Preclinical Evidence. ↑ Increased expression/level, ↓ decreased expression/level, and ↔ expression/level unchanged.

Target cytokines	Study type/Trial number	Intervention detail/sample size	Internation duration	Outcome in NAFLD	Expression level of cytokines	Adverse effect/efficacy limitation	Ref
IL-1β and TNF-α	Clinical/ChiCTR1900024866	2.34 g/day of eicosapentaenoic acid (EPA) + docosahexaenoic acid (DHA) + 1680 IU vitamin D3/111 patients	3 months	The groups supplemented with exerted beneficial effects on markers of hepatocellular damage and plasma triacylglycerol levels in subjects with NAFLD, and also showed additional benefits on insulin resistance (IR) and inflammation	↓	There were no reported side effects or adverse events associated with any of the supplement treatments. However, the main limitations of this trial include the absence of a vitamin D-only control group, imbalances in baseline triglyceride levels between groups, and a short duration with a modest sample size	Guo et al. (2022)
TNF-α and TGF-β1	Clinical/IRCT.ir (IRCT 2012071810333N1)	50,000 International Unit (IU) (vitamin D)/53 patients	4 months	Vitamin D supplementation in patients with NAFLD has been shown to help reduce oxidative stress and inflammation	↔	No adverse effects were reported in this study; however, the vitamin D treatment does not appear to significantly improve liver enzymes, IR, or steatosis grade within 4 months	Sharifi et al. (2014)
TNF-α	Clinical	600,000 IU (vitamin D)/81 patients	6 months	Vitamin D may improve bile acid metabolism, reduce oxidative stress, and modulate adipocytokines (e.g., increase adiponectin)	↓	No side effects were reported; however, there were no significant changes in weight, BMI, or HOMA-IR, indicating the benefits were independent of weight loss or IR.	Sakpal et al. (2017)
TNF-α and IL-10	Preclinical	0.1 µM (vitamin D)/not mentioned	6 weeks	The experimental results in zebrafish confirmed the significant benefits of vitamin D, including reductions in hepatic lipid deposition, improved antioxidant defenses, decreased oxidative stress, and reduced apoptosis	↓	No adverse effects were reported in this study	Dharshan et al. (2025)
TNF-α, IL-10, or TGF-β	Clinical/(EudraCT, Ref. 2005–000860–24)	60 IU (vitamin E)/179 patients	12 months	A 12-month Realsil treatment (silybin, phosphatidylcholine, low-dose vitamin E) safely improves liver enzymes, IR, and histological features of NAFLD, including fibrosis	↔	Mild, transient adverse events (diarrhea, dysgeusia, pruritus, abdominal pain) occurred, with no serious drug-related events The effects of vitamin E alone cannot be isolated, as evidenced by several factors: only 35 of 138 patients underwent a second biopsy, the study included small subgroups of patients with hepatitis C virus/advanced NASH, and there was a high dropout rate (41 patients). Additionally, baseline NAS imbalance was present, although it was statistically adjusted	Loguercio et al. (2012)
TNF-α	Clinical	Vitamin E 400 IU/67 patients	6 months	Improved insulin sensitivity, reduced triglycerides, reduced BMI, improved steatosis	↔	Vitamin E was well tolerated; no adverse effects were reported. However, it did not significantly affect inflammatory cytokines (TNF-alpha, adiponectin). The study was limited by its short duration, lack of histological confirmation, and small sample size (n = 67 participants)	Akcam et al. (2011)
TNF-α and IL-6	Clinical/NCT02962297	Vitamin E 300 mg/124 patients	96 weeks	A daily dose of 300 mg of Vitamin E administered over 96 weeks was found to be a safe and effective treatment for non-diabetic MASH patients, leading to significant improvements in liver histology and a reduction in markers of inflammation and apoptosis	↓	The study reported 11 adverse events (7 vitamin E, four placebo) and 12 serious events, but none were treatment-related; no cardiovascular events, stroke, prostate cancer, or new-onset diabetes occurred	Song et al. (2025)
TNF-α and IL-6	Clinical/NCT02962297	vitamin E 300 mg/120 patients	24 weeks	Histological improvement (NAFLD activity score ↓≥2, ballooning improvement, no fibrosis worsening) and biochemical improvement: ALT, AST, lipid profile, glucose metabolism	Trial results are in progress for cytokines	The Nondiabetic Patients with Nonalcoholic Steatohepatitis (VENS) trial represents the first randomized controlled trial in China assessing the efficacy of vitamin E in nondiabetic NASH patients, with particular emphasis on the haptoglobin genotype. Results are not yet available, but the study is designed to assess histological, biochemical, and quality-of-life outcomes. Limitations include the relatively low dose of vitamin E and exclusion of diabetic and morbidly obese patients	Zang et al. (2018)
TNF-α	Clinical/NCT04198805	Vitamin E 1000 mg/200 patients	6 months	The 6-month treatment with vitamin E, DHA, or their combination was not efficacious in reducing liver fat or improving any other biomarkers of MASLD in the studied population	↔	Adverse events were generally mild to moderate and comparable across treatment and placebo groups, with gastrointestinal symptoms such as diarrhea, constipation, abdominal pain, and nausea being the most common. One case of probable drug-induced liver injury occurred in the DHA group, characterized by asymptomatic elevation of liver enzymes at 3 months, which resolved after discontinuation of treatment; it was adjudicated as “probable” but did not meet Hy’s Law criteria. The study had several limitations, including the absence of liver biopsy, which prevented assessment of histologic features such as inflammation, ballooning, and fibrosis; the use of fixed dosages that may not have been optimal; the relatively short 6-month duration that may have been insufficient to capture treatment effects, particularly histologic changes; and limited statistical power for secondary analyses, especially in the vitamin E-alone and DHA-alone groups	Alkhouri et al. (2024)
TNF-α and IL-1β	Preclinical	Vitamin E (α-Tocopherol) 0.01%/not mentioned	8 weeks	Improves histology and oxidative stress, but is less effective against tumors	↔	DMT-1 expression and hepatic ferritin levels were elevated, indicating iron dysregulation, alongside unfavorable gut microbiome shifts (↓ Lactobacillales, ↑ Prevotella). Moreover, it showed lower efficacy than L-carnitine in suppressing hepatocarcinogenesis	Ishikawa et al. (2014)
TNF-α and IL-6	Preclinical	Vitamin B9 (Folic Acid) 26 mg/kg/not mentioned	8 weeks	Folic acid supplementation was effective in minimizing both hepatic lipid accumulation and the aggregation of inflammatory foci in animal subjects maintained on a high-fat diet.	↓	Crucially, the study reported no adverse side effects from folic acid supplementation at the dose used. Folic acid did not affect body weight gain caused by the HFD. It improved metabolic health (liver-specific outcomes) without causing weight loss or any other noted adverse effects in the mice	Sid et al. (2018)
IL-22, IL-8 and TNF-α	Preclinical	Vitamin B9 (Folic Acid) (50, 100, and 150 mg/kg/50 rats	8 weeks	Folate in improving liver enzyme function and the production of immune mediators in NAFLD	At 150 mg/kg treatment, the IL-8 and TNF-α ↓, while IL-22 ↑	The study reported no adverse side effects from folate supplementation at any of the doses used. The treatment was well tolerated, and all observed changes were beneficial	Youssry and Kamel (2019)

Compelling evidence establishes that VD exerts potent immunomodulatory effects in NAFLD and NASH. Its primary mechanism involves reprogramming the hepatic immune environment by suppressing pro-inflammatory cytokines (e.g., TNF-α, IL-6) and promoting anti-inflammatory signals (e.g., IL-10). This is primarily achieved through the activation of VDR signaling, which inhibits key pathways, such as NF-κB, promotes a shift in macrophage polarization from the pro-inflammatory M1 to the anti-inflammatory M2 state, and directly suppresses HSC activation to reduce fibrosis. Future research must transition these robust preclinical findings into clinical validation. Future research must validate these benefits in large-scale human trials to confirm that VD supplementation leads to significant histological improvements in liver inflammation and fibrosis, not just biomarker changes.

#### 3.2.2 Vitamin E (VE) and its effects on cytokines

Clinical evidence also supports the anti-inflammatory potential of VE in NASH, primarily through the modulation of key cytokines. Alongside its antioxidant role, VE has demonstrated significant immunomodulatory properties by influencing the production and release of various cytokines central to NAFLD pathogenesis. In a randomized controlled trial conducted in Egyptian patients, supplementation with VE significantly decreased circulating levels of the pro-inflammatory cytokine IL-6 and the chemokine MCP-1, with reductions of approximately 57% and 55%, respectively. These effects were more pronounced than those observed with ursodeoxycholic acid (UDCA) or pentoxifylline (PTX), highlighting the superior efficacy of VE in attenuating inflammation. Moreover, the observed correlation between the decline in serum ALT and reductions in IL-6 and MCP-1 suggests that the antioxidant properties of VE may contribute to dampening the inflammatory cascade through the mitigation of oxidative stress (Fouda et al., 2021). Likewise, a randomized clinical trial in obese children with NAFLD demonstrated that supplementation with a tocotrienol-rich fraction (TRF) of VE exerted significant anti-inflammatory effects. TRF treatment markedly downregulated the gene expression of key pro-inflammatory cytokines, including IL-6 and TNF-α, compared to baseline, whereas no such changes were observed in the placebo group. These findings underscore the potent immunomodulatory capacity of tocotrienol-based VE and suggest its potential in attenuating inflammatory pathways that drive NAFLD pathogenesis in the pediatric population (Al-Baiaty et al., 2024).

Expanding this evidence, other combination therapies incorporating VE have shown promise. 2019 clinical research examined the impact of VE and alpha-linolenic acid (ALA) supplementation on inflammatory markers and liver enzymes in obese patients with NAFLD, a disorder closely associated with inflammation caused by obesity. Forty-five participants received either a placebo or the ALA and VE combination during the 12-week study (Hosseinpour-Arjmand et al., 2019). When compared to a placebo, ALA with VE supplementation significantly raised serum adiponectin levels while lowering IL-6 and insulin levels. When compared to their baseline values, the liver steatosis grade of both the ALA plus VE-treated group and the placebo group showed a significant improvement; however, the changes in the two groups were not statistically different. The trial’s findings showed that daily ALA with VE supplementation was well tolerated and had no negative side effects.

Further supporting its role in cytokine modulation, VE has been shown to impact pro-fibrotic signaling. In a prior pilot study by Hasegawa et al. (2001), VE (α-tocopherol) supplementation for 1 year significantly reduced plasma levels of the pro-fibrotic cytokine TGF-β1 in patients with NASH. This reduction in TGF-β1, a key mediator of hepatic fibrosis, was accompanied by improvements in liver enzymes and histology, suggesting that VE’s efficacy in NASH may be partly mediated through the suppression of this pathogenic cytokine. Likewise, in a recent pediatric NAFLD trial by Mosca et al. (2021) (Figure 3B), a combination of VE and hydroxytyrosol (HXT) significantly modulated the systemic inflammatory profile. While levels of IL-1β and TNF-α decreased in both the treatment and placebo groups (likely due to lifestyle interventions), only the HXT + VE group exhibited a significant increase in the anti-inflammatory cytokine IL-10 and a decrease in the pro-inflammatory cytokine IL-6. The rise in IL-10 was positively correlated with the treatment’s antioxidant compounds and negatively correlated with oxidative stress markers, indicating that VE’s contribution to the anti-inflammatory effect is mediated through the enhancement of anti-inflammatory pathways and the reduction of oxidative damage.

The anti-fibrotic effects of VE combinations also imply an indirect role in cytokine regulation. Panera et al. (2022) (Figure 3C) investigated the therapeutic potential of combined HXT and VE supplementation in NAFLD, with a particular focus on fibrosis. Although cytokine modulation was not the primary endpoint, the study demonstrated that this treatment strategy exerted anti-fibrotic effects predominantly through inhibition of the TGF-β/SMAD signaling axis. Specifically, the combination significantly reduced TGF-β-induced ROS generation in HSCs and attenuated the nuclear translocation of SMAD2/3, key mediators of pro-fibrogenic and inflammatory signaling. While a comprehensive cytokine profile was not assessed, the blockade of this central pathway implies an indirect suppression of TGF-β–driven pro-inflammatory cytokine cascades, thereby contributing to the overall attenuation of hepatic fibrosis. Moreover, in a recent study, VE was administered as part of a combined supplement (RealSIL 100D®) alongside silybin and VD. The treatment significantly reduced pro-inflammatory cytokines, including TNF-α, TGF-β, IL-18, and IL-22, after 6 months compared to untreated controls, suggesting that VE contributes to the anti-inflammatory effects observed (Federico et al., 2019). However, these improvements were not sustained after treatment cessation, indicating that the cytokine modulation is dependent on continued supplementation. The effect was particularly pronounced in patients with concurrent metabolic syndrome, highlighting a synergistic role for VE in mitigating inflammation in NAFLD.

Contrary to these findings, several studies report a more limited immunomodulatory role for VE in NASH. In a recent randomized controlled trial, VE did not produce a significant reduction in key inflammatory cytokines such as TNF-α or NLRP3 in patients with NASH, in contrast to the significant decreases observed in the N-acetyl cysteine and rosuvastatin groups. While VE effectively reduced oxidative stress markers like malondialdehyde, its anti-inflammatory impact on cytokine levels was negligible, underscoring its limited role in modulating the inflammatory pathways central to NASH progression. These findings suggest that VE’s primary benefit in NASH may be through antioxidant mechanisms rather than direct cytokine suppression (Zakaria et al., 2025). Similarly, a previous preclinical study of VE supplementation did not significantly alter plasma levels of the pro-fibrotic cytokine TGF-β1 or the adipocytokine leptin compared to the cholesterol-fed control group. However, it also did not significantly increase levels of the protective adipokine adiponectin, an effect which was uniquely observed with the Kampo formula KBG (Fujimoto et al., 2008). These findings suggest that while VE ameliorated oxidative stress and lipid profiles, its impact on key cytokines implicated in NASH pathogenesis was limited in this non-obese rabbit model. Collectively, these findings highlight the complex and context-dependent role of VE in modulating the inflammatory environment of NAFLD.

The role of VE in cytokine modulation during NAFLD is complex and context-dependent. Evidence from clinical studies suggests that VE, either alone or in combination with other compounds, can significantly reduce pro-inflammatory cytokines such as IL-6, TGF-β1, and MCP-1, while enhancing anti-inflammatory mediators including IL-10 and adiponectin. These effects are strongly linked to its antioxidant activity, where lowering oxidative stress attenuates downstream inflammatory signaling. Genetic variations, such as the Hp genotype, may further influence therapeutic outcomes. However, its impact is not universal—some studies report limited or inconsistent effects, particularly on TNF-α, raising the possibility that its benefits derive more from indirect antioxidant mechanisms than from direct cytokine modulation. VE shows beneficial effects in NAFLD by reducing oxidative stress and regulating cytokine pathways, with supportive evidence from clinical and preclinical studies (Table 3). Future research should prioritize large-scale, genotype-stratified trials, explore pathways like NLRP3 inflammasome and TGF-β/SMAD signaling, and determine whether combination therapies provide superior efficacy compared to monotherapy.

#### 3.2.3 Vitamin B (VB) and its effects on cytokines

VB includes eight different kinds of substances that are important cofactors in many enzyme reactions and have a role in both anabolism and catabolism (Hanna et al., 2022). Thiamine (VB1), a necessary cofactor for α-keto acid decarboxylase and transketolase, is important for mitochondrial activity, oxidative stress control, and hepatic glucose and lipid metabolism (Kalyesubula et al., 2021). In sheep, high-dose thiamine supplementation alleviated overnutrition-induced hepatic steatosis by reducing IL-8 expression in leukocytes and hepatic TNF levels, while having little effect on IL-1β, MCP-1, or interferon-gamma IFN-γ (Kalyesubula et al., 2021). These findings suggest a selective anti-inflammatory action, indicating that thiamine may help counteract diet-induced inflammation associated with metabolic liver disease.

Beyond thiamine, other B vitamins also demonstrate significant immunomodulatory properties in the context of liver disease. VB3, in the form of nicotinamide (NAM), demonstrates therapeutic potential in managing NAFLD. In a recent study, NAM supplementation significantly reduced hepatic levels of the pro-inflammatory cytokine TNFα in a rat model of carbohydrate-induced steatosis. This anti-inflammatory effect was mechanistically linked to NAM’s ability to improve the hepatic redox environment, evidenced by a more reduced glutathione redox potential (GSH/GSSG Eh) and decreased oxidative stress, positioning it as a modulator of inflammation in metabolic disease (Mejía et al., 2018). Likewise, in an *in vitro* study, niacin (VB3) significantly reduced the secretion of the pro-inflammatory cytokine IL-8 from human hepatocytes (HepG2 and primary cells) that were stimulated with palmitic acid. This anti-inflammatory effect was mechanistically linked to niacin’s ability to attenuate oxidative stress by inhibiting NADPH oxidase activity and reducing ROS production, a key driver of inflammatory signaling in NAFLD pathogenesis (Ganji et al., 2015).

However, the anti-inflammatory role of VB3 is not universally observed across all experimental models, as evidenced by a controversial study by Ganji et al. (2014) Niacin supplementation in a HFD rat model of NAFLD did not significantly modulate pro-inflammatory cytokine expression. Specifically, hepatic mRNA levels of TNF-α, a prototypic inflammatory cytokine, were unchanged compared to both control and HFD groups. This lack of effect on cytokine expression occurred despite niacin’s demonstrated efficacy in preventing and regressing hepatic steatosis and reducing oxidative stress. Therefore, the study concludes that the therapeutic benefits of niacin in this model are mediated through mechanisms independent of cytokine modulation, namely, the robust inhibition of hepatic DGAT2 activity and a reduction in lipid peroxidation products.

**FIGURE 4:**
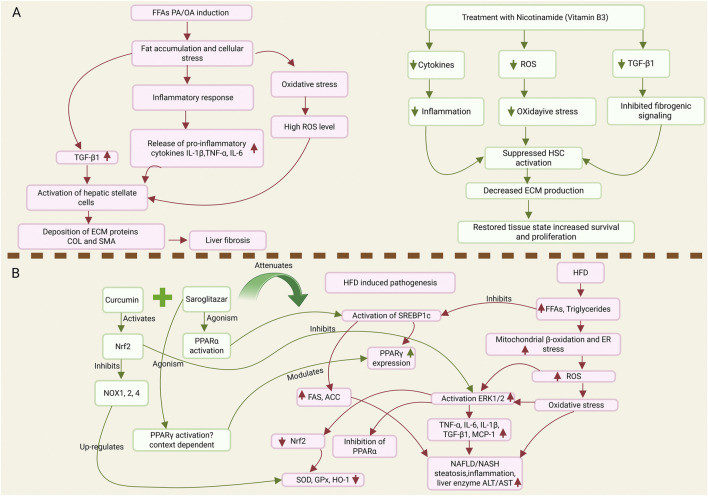
Targeting multimodal pathways in NAFLD, nicotinamide suppresses inflammation, oxidative stress, and fibrosis while saroglitazar and curcumin restore antioxidant defenses and lipid homeostasis. **(A)** Multimodal mechanism of Nicotinamide (NA) in alleviating NAFLD-pathology in a multicellular microtissue model. Induction of fibrosis with free fatty acids (FFAs: palmitic/oleic acid) causes lipid accumulation and cellular stress. This stress triggers three parallel pathogenic pathways: (1) an inflammatory response, releasing cytokines (IL-1β, IL-6, TNF-α); (2) a surge in reactive oxygen species (ROS); and (3) upregulation of the fibrogenic cytokine TGF-β1. These pathways converge to activate HSCs, resulting in the excessive deposition of extracellular matrix (ECM) proteins (Collagen I and α-SMA) and the progression of fibrosis. Treatment with Nicotinamide (NA) simultaneously targets all three pathways. NA reduces the levels of pro-inflammatory cytokines, scavenges ROS to alleviate oxidative stress, and downregulates TGF-β1 expression. By mitigating these key drivers, NA effectively suppresses HSC activation, leading to a significant decrease in ECM production and a promotion of microtissue health and survival. **(B)** Proposed mechanism of action for saroglitazar and curcumin in ameliorating HFD-induced NAFLD/NASH. Consumption of a High-Fat Diet (HFD) leads to an influx of free fatty acids (FFAs) and hepatic triglyceride accumulation. This promotes mitochondrial β-oxidation and ER stress, resulting in excessive Reactive Oxygen Species (ROS) production. ROS induces oxidative stress, which suppresses Nrf2 activity (reducing antioxidant enzymes like SOD, GPx, and HO-1) and activates the pro-inflammatory ERK1/2 pathway. ERK1/2 activation increases pro-inflammatory cytokines (TNF-α, IL-6, IL-1, TGF-β1) and inhibits PPARα. Concurrently, FFAs activate SREBP1c, which upregulates lipogenic genes (FAS and ACC) and PPARγ, thereby further promoting fat storage. These processes ultimately lead to NAFLD/NASH. Saroglitazar (a PPARα/γ agonist) and curcumin act by activating PPARα and Nrf2 pathways. PPARα activation inhibits SREBP1c, reducing lipogenesis. Nrf2 activation boosts antioxidant defenses, suppresses ERK1/2 and NOX enzymes, and reduces oxidative stress and inflammation. The combined action of these drugs effectively counteracts the key pathological mechanisms of NAFLD.

Further supporting a role for VB3, recent evidence continues to highlight the therapeutic potential of nicotinamide. Using a multicellular disease model, the study demonstrated that nicotinamide exerts robust anti-inflammatory effects through modulation of cytokine expression. Specifically, treatment with nicotinamide significantly reduced the levels of key pro-inflammatory cytokines, including TNF-α, IL-1β, and IL-6, alongside a marked suppression of TGF-β1, a central mediator of fibrogenesis (Figure 4A) (Sani et al., 2025). The attenuation of the TGF-β1 signaling axis was proposed as a significant mechanism underlying the anti-fibrotic actions of nicotinamide, positioning it as a promising candidate for targeting the inflammatory and fibrotic pathways that drive disease progression in NAFLD.

The anti-inflammatory potential of B vitamins extends beyond VB3 to include folate (VB9). In a rat model of HFD-induced MASLD, serum levels of IL-22 were markedly reduced, while the hepatic expression of the autophagy marker LC3B was increased. Supplementation with folate (VB9) significantly attenuated hepatic inflammation in a dose-dependent manner, as evidenced by the downregulation of pro-inflammatory mediators, including TNF-α, IL-8, and LC3B. Interestingly, increasing doses of folate not only reduced autophagy in hepatocytes but also restored IL-22 levels, suggesting a potential interplay between autophagy and IL-22 signaling in mediating hepatoprotection (Youssry and Kamel, 2019).

The mechanism by which folate exerts its protective effects against MASLD appears to be multifaceted. An HFD promotes hepatic lipid accumulation, triggers the activation of the pro-inflammatory transcription factor NF-κB, and upregulates the expression of inflammatory genes. Folate supplementation counteracts these effects by inhibiting NF-κB activation, which dramatically lowers the levels of inflammatory cytokines such as IL-6 and TNF-α, thereby reducing liver inflammation (Sid et al., 2018). Furthermore, folate’s antioxidant properties help mitigate pro-oxidant-induced oxidative stress and mitochondrial dysfunction (Chang et al., 2007). Through these combined mechanisms—reducing lipid accumulation, suppressing inflammation, and combating oxidative stress—folate supplementation helps mitigate the progression of MASLD.

This mechanism is further supported by detailed *in vitro* research. Bagherieh et al. (2023) examined the anti-inflammatory effects of folic acid in an *in vitro* NAFLD model using palmitate-treated HepG2 cells, which mimic lipid overload and metabolic stress typical of early disease. Palmitate exposure induced a strong inflammatory response, while folic acid supplementation significantly reduced the expression of key cytokines (TNF-α, IL-6, IL-1β). Mechanistically, folic acid lowered intracellular homocysteine levels, decreased ROS generation, and inhibited NF-κB activation, thereby dampening the pro-inflammatory cascade. These findings suggest that folic acid may serve as a promising nutritional strategy to alleviate hepatocellular inflammation and slow NAFLD progression. B vitamins, especially folate (B9), improve NAFLD by modulating cytokines, with supporting evidence from preclinical studies (Table 3).

Based on the collective evidence, B vitamins—particularly thiamine (B1), nicotinamide (B3), and folate (B9)—demonstrate significant potential as protective nutritional strategies against NAFLD progression. Their efficacy stems from a multi-targeted mechanism: they ameliorate hepatic steatosis, potently reduce oxidative stress by scavenging ROS and improving redox status, and directly inhibit the activation of the central NF-κB inflammatory pathway. This action collectively suppresses the production of key pro-inflammatory cytokines (TNF-α, IL-6, IL-1β) and profibrotic mediators like TGF-β1. Future protective strategies should prioritize clinical trials to validate the therapeutic and preventive efficacy of B vitamin supplementation, either individually or in combination, for mitigating inflammation and fibrosis in human NAFLD, potentially offering a simple dietary intervention to slow disease progression.

### 3.3 Curcumin and its effects on cytokines

Curcumin, the primary bioactive compound in turmeric (*Curcuma longa*), has been widely studied. More recently, clinical research has also explored the efficacy of a formulation combining curcumin with piperine. Panhai and coworkers investigated the efficacy of 500 mg of curcumin plus 5 mg of piperine (Curcumin C3 Complex® and Bioperine®, Sami Labs Ltd.) in adult individuals with NAFLD. For 12 weeks, seventy adults received one capsule after meals and were randomly allocated to either the treatment or placebo group. Findings indicated that, in comparison to a placebo, the administration of Curcumin C3 Complex® and Bioperine® improved the liver and lipid profile without changing hematological parameters, hence reducing the severity of NAFLD (Panahi et al., 2019). The same research team also examined the formulation’s impact of Curcuminoids (comprising curcumin) on serum inflammatory markers, specifically TNF-α and IL-6, in a randomized, double-blind, placebo-controlled study involving 55 individuals with NAFLD. After 8 weeks of dietary supplementation, a reduction in serum cytokine levels was observed. This suggests that lowering these cytokines may be one mechanism through which curcuminoids exert their anti-steatotic effect (Saberi-Karimian et al., 2020).

Beyond these direct anti-inflammatory effects on the liver, emerging evidence highlights a novel gut-centric mechanism by which curcumin mitigates hepatic inflammation. Liao et al. (2025) demonstrated that curcumin’s protective effect against visceral adiposity in a rat model of metabolic dysfunction-associated steatohepatitis (MASH) operates through the gut-hormone axis. Their work showed that curcumin alleviates hypoxia-induced damage to the intestinal epithelial and vascular barriers. This protection, in turn, suppresses the release of gastric inhibitory polypeptide (GIP) from the small intestine. By inhibiting GIP, curcumin reduced the activation of GIP receptors in perirenal adipose tissue, ultimately attenuating local adipogenesis and inflammation (e.g., reduced TNF-α and IL-1β). This study provides a direct mechanistic link between curcumin, the integrity of the intestinal barrier, a key gut hormone, and the regulation of adipose tissue inflammation, which is a critical driver of systemic metabolic disease and hepatic injury.

Furthermore, the therapeutic scope of curcumin has been expanded to include the dual modulation of key hepatocellular death pathways, providing a direct link to the control of cytokine-driven inflammation. Eldken et al. (2025) provide compelling evidence for this in a high-fat, high-fructose diet model of metabolic dysfunction-associated steatotic liver disease (MASLD). Their findings indicate that curcumin’s hepatoprotective effect is not limited to a single pathway. Instead, it simultaneously promotes autophagy (evidenced by increased *BECN1* gene expression and decreased p62 protein accumulation) while suppressing pyroptosis (evidenced by reduced hepatic levels of Gasdermin D and cleaved caspase-1, alongside decreased TNF-α and IL-1β). This dual regulation is particularly significant because of the well-established crosstalk between these processes. Impaired autophagy is known to exacerbate the activation of the NLRP3 inflammasome, the master complex that triggers pyroptotic cell death. When a hepatocyte undergoes pyroptosis, its membrane ruptures, releasing potent pro-inflammatory cytokines—including IL-1β and TNF-α—directly into the surrounding tissue, thereby amplifying local and systemic inflammation. By simultaneously restoring autophagic flux and inhibiting the pyroptotic machinery, curcumin effectively breaks this vicious cycle. This mechanism highlights a sophisticated strategy for mitigating liver damage that operates at the very intersection of cell survival and inflammatory signaling, offering a powerful means to control the cytokine cascade central to NASH pathogenesis.

Furthermore, pre-clinical research has designed novel compounds to enhance this anti-inflammatory effect. Wu et al. (2019) reported that the resveratrol–curcumin hybrid compound a19, designed to integrate the pharmacological advantages of curcumin, exerts strong anti-inflammatory activity in the context of NAFLD. In both *in vivo* and *in vitro* experimental systems, administration of a19 markedly suppressed the mRNA expression of pivotal pro-inflammatory cytokines, including TNF-α, IL-6, and IL-1β. This effect was mechanistically linked to the inhibition of the extracellular signal-regulated kinase (ERK) signaling pathway, a central regulator of inflammatory cascades. The authors emphasized that modulation of cytokine production represents a key mechanism underlying the hepatoprotective properties of a19, highlighting its potential as a therapeutic candidate for managing NAFLD-associated inflammation and injury.

Further elucidating the molecular mechanisms, other studies have identified specific inflammatory pathways targeted by curcumin. Afrin et al. (2017a) demonstrated that curcumin exerts a profound immunomodulatory effect in a NASH-HCC mouse model through inhibition of the high mobility group box 1 (HMGB1)–NF-κB signaling axis. Curcumin treatment markedly reduced the cytosolic translocation of the pro-inflammatory damage-associated molecular pattern (DAMP) HMGB1, thereby limiting the subsequent nuclear translocation and activation of NF-κB. This suppression of the central inflammatory pathway resulted in a significant downregulation of pro-inflammatory mediators, including IL-1β, IFN-γ, and interferon-γ-inducible protein-10 (IP-10). These findings indicate that the hepatoprotective action of curcumin in NASH-associated hepatocarcinogenesis is primarily mediated through the inhibition of HMGB1-driven cytokine and chemokine cascades. Consistently, (Afrin et al., 2017b) further reported that curcumin alleviates extrahepatic complications of NASH, particularly chronic kidney injury, by attenuating renal inflammation. In this model, curcumin treatment significantly decreased the elevated protein levels of TNF-α, IL-1β, and IFN-γ in kidney tissue. Mechanistically, these renoprotective effects were linked to the suppression of endoplasmic reticulum (ER) stress and downstream MAPK signaling pathways, including JNK and ERK1/2, both of which drive inflammatory gene expression. Together, these studies underscore curcumin’s capacity to modulate central inflammatory signaling pathways across multiple organs, thereby conferring both hepatoprotective and renoprotective benefits in the setting of NASH.

The anti-inflammatory properties extend to the individual components of curcumin itself. In a recent study, curcumin, demethoxycurcumin, and bisdemethoxycurcumin significantly attenuated the production of pro-inflammatory cytokines in a MCD diet-induced NAFLD mouse model. All three curcuminoids suppressed the MCD diet-induced upregulation of serum IL-6 and TNF-α, with bisdemethoxycurcumin demonstrating the most potent effect, reducing cytokine levels to those of the normal control group. This anti-inflammatory response was mechanistically linked to the curcuminoids’ ability to inhibit the expression of toll-like receptors, toll-like receptor-2 (TLR-2) and TLR-4 at both the mRNA and protein levels, which are upstream regulators of NF-κB and MAPK signaling pathways that drive cytokine production (Lee et al., 2022).

A synergistic approach combining natural products and targeted small molecules may offer a more effective strategy for addressing the multifactorial pathogenesis of NAFLD. This is illustrated by the actions of a curcumin derivative, Curcumin5-8, which activates AMPK and regulates autophagy to ameliorate fatty liver, and the small-molecule inhibitor EW-7197, which blocks the TGF-β receptor I to mitigate fibrosis and oxidative stress *via* the SMAD2/3 pathway. Their combined application in murine NASH models and cultured fibrotic hepatocytes resulted in the attenuation of both fibrosis and steatohepatitis, successfully harnessing the benefits of both therapeutic modalities (Ha et al., 2023).

The anti-inflammatory mechanism of curcumin has been further elucidated, revealing its action on specific molecular pathways. In an HFD-induced NAFLD/NASH rat model, curcumin and saroglitazar effectively reduced pro-inflammatory cytokines (TNFα, IL-1β, IL-6, TGF-β1, MCP-1, and COX-2) by suppressing ERK1/2 signaling while activating the Nrf2 antioxidant pathway. This dual action attenuated oxidative stress and inflammation, leading to improved hepatic pathology (Figure 4B) (Afarin et al., 2024). While these preclinical results are promising, clinical studies are needed to confirm the cytokine-modulating effects of the curcumin and saroglitazar combination in humans with NASH. Additionally, a previous study in obese murine models with hepatic steatosis has shown that curcumin exhibits potent anti-inflammatory effects. Treatment with curcumin significantly reduced both the expression and systemic release of key pro-inflammatory mediators, including TNF-α, IL-6, and MCP-1, in a dose-dependent manner. These effects were associated with decreased adipose tissue mRNA levels and lower circulating cytokine concentrations. Mechanistically, curcumin attenuated hepatic inflammation by limiting macrophage infiltration, as evidenced by reduced accumulation of F4/80-positive cells, and by suppressing NF-κB activity, a central regulator of inflammatory gene transcription (Kuo et al., 2012). Through this coordinated regulation of cytokine production and signaling, curcumin contributed to an improved inflammatory milieu and alleviation of obesity-induced hepatic injury. Moreover, curcumin significantly inhibited M1 macrophage activation in a methionine and MCD diet-induced NASH animal model, which decreased the expression of the pro-inflammatory cytokines IL-1β and TNF-α and alleviated hepatic inflammation and liver damage in NASH (Tong et al., 2021). Furthermore, according to Cunningham et al. (2018), curcumin administration was effective in lowering serum indicators linked to liver injury, hepatic steatosis, NAFLD activity scores, and hepatocellular inflammation. The treatment of curcumin considerably reduced hepatic inflammation (TNF-α) and improved the NASH phenotype in female Wistar rat models. Curcumin may be able to prevent NASH in adolescent rats fed a high-fructose diet by boosting AMPKα expression and lowering TNF-α expression in neonatal rats (Ibrahim et al., 2019).

**FIGURE 5:**
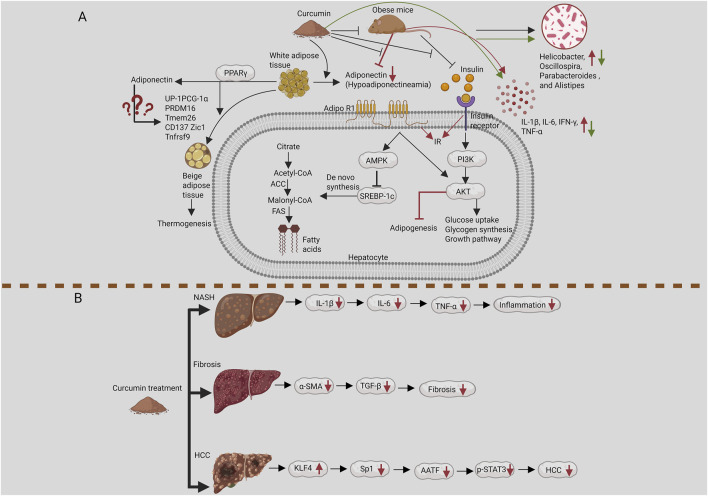
Protective mechanisms of curcumin in metabolic liver disease. Curcumin modulates gut dysbiosis, adipose browning, and insulin pathways while suppressing antagonizing transcription factor (AATF)-driven steatohepatitis and hepatocellular carcinoma (HCC). **(A)** Schematic representation of curcumin’s effects on obesity-induced dysbiosis, NAFLD, and adipose tissue browning. In mice with high-fat diet (HFD)-induced obesity, body weight, epididymal fat mass, and inflammation were elevated. Curcumin supplementation alleviated HFD-related dysbiosis and hepatic steatosis. Mechanistically, curcumin enhanced adiponectin and adiponectin receptor-1 expression, improved insulin signaling activity, and suppressed lipogenesis-associated factors. These findings highlight the potential of curcumin in modulating gut dysbiosis, lipid metabolism, and insulin pathways in the context of diet-induced obesity. **(B)** Molecular mechanisms of curcumin in counteracting AATF-driven metabolic dysfunction, steatohepatitis, and HCC. Curcumin was administered for 12 weeks to mice that were fed either a western diet with sugar water (WDSW)/CCl_4_ or a chow diet with normal water (CDNW)/CCl_4_. In WDSW/CCl_4_ mice, curcumin treatment decreased fibrosis, NASH, and the development of HCC. Mechanistically, curcumin decreased apoptosis and AATF expression by altering the Kruppel-like factor 4 (KLF4) and specificity protein 1 (Sp1) signaling pathways.

Recent research continues to refine our understanding of how curcumin modulates inflammation and metabolism. In a recent study, Curcumin has demonstrated potent immunomodulatory activity by attenuating obesity-induced metaflammation in experimental models of NAFLD. In HFD-induced NAFLD mice, curcumin downregulates IL-6, IL-1β, TNF-α, and IFN-γ expression in hepatic and adipose tissues, thereby dampening macrophage-driven inflammation and apoptotic signaling (Chiu et al., 2025). Mechanistically, curcumin suppresses NF-κB activation by preventing IκB-α degradation while enhancing adiponectin/adiponectin receptor-1 signaling, which restores phosphatidylinositol 3-kinase (PI3K)/protein kinase B (AKT) activity and improves insulin sensitivity. This signaling cascade inhibits lipogenic transcriptional programs mediated by SREBP-1 and its downstream enzymes (FASN, ACC, SCD-1), thus reducing hepatic triglyceride accumulation. Concurrently, curcumin stimulates PPARγ and PGC-1α, driving uncoupling protein-1 (UCP-1)–dependent browning of white adipose tissue, which enhances thermogenesis and contributes to the resolution of chronic inflammation (Figure 5A) (Chiu et al., 2025). Collectively, evidence from NAFLD models highlights curcumin as a metabolic regulator that links suppression of pro-inflammatory cytokines to improved lipid homeostasis and insulin signaling. In addition, in experimental models of MASH and HCC, curcumin administration significantly attenuated the disease-associated elevation of key pro-inflammatory cytokines, including IL-1β, IL-6, and TNF-α, in both hepatic tissue and systemic circulation. This cytokine suppression was mechanistically linked to the downregulation of stress-responsive kinases (ERK1/2, JNK), reduced macrophage infiltration, and inhibition of the central inflammatory regulator NF-κB. Consequently, curcumin disrupts critical pro-oncogenic signaling pathways, including those mediated by STAT3 and AATF. Through these coordinated actions, curcumin not only attenuates the inflammatory milieu that drives fibrosis and oncogenesis but also directly contributes to its observed hepatoprotective and anti-tumorigenic effects (Figure 5B) (Srinivas et al., 2024). Collectively, curcumin’s cytokine-modulatory properties contribute significantly to its hepatoprotective and anti-tumorigenic effects. Curcumin improves NAFLD by modulating cytokines, with evidence from clinical and preclinical studies (Table 4).

**TABLE 4:** The Role of Curcumin in NAFLD *via* Cytokine Modulation Based on Clinical and Preclinical Evidence. ↑ Increased expression/level, ↓ decreased expression/level, and ↔ expression/level unchanged.

Target cytokines	Study type/Trial number	Intervention detail/sample size	Internation duration	Outcome in NAFLD	Expression level of cytokines	Adverse effect/efficacy limitation	Ref
TNF-α	Clinical/IRCT20100524004010N24	1,500 mg/50 patients	12 weeks	The results indicated that curcumin supplementation significantly reduced hepatic fibrosis from baseline measurements. Hepatic steatosis and serum liver enzyme levels were also reduced	↓	Curcumin was found to be safe and well-tolerated, with no adverse effects reported by any of the participants. This specific formulation and dosage showed an excellent safety profile in this 12-week clinical trial	Saadati et al. (2019)
IL-1β, TNF-α and IL-6	Clinical/ISRCTN70887063	667 mg/140 patients	12 weeks	After treatment for NAFLD, patients exhibited reduced liver fat, improved steatosis grade, enhanced insulin sensitivity, and increased antioxidant capacity, indicating improved liver health and metabolic function	↔	The nutraceutical was well-tolerated, with all reported adverse events (AEs) classified as Grade 1 (mild). The most common AEs were gastrointestinal in nature, such as discomfort and diarrhea. Notably, fewer participants in the nutraceutical group reported abdominal discomfort compared to the placebo group (7 vs. 13 participants). Furthermore, no notable alterations in blood pressure, creatinine, glucose, lipid profiles, or liver enzyme levels were detected, suggesting a lack of toxic effects	Ferro et al. (2022)
IL-1β, TNF-α and IL-6	Clinical/(TCTR20140303003)	1,500 mg/78 patients	12 months	Curcumin treatment in NAFLD resulted in significant reductions in liver fat, stiffness, glycemic markers, body fat, BMI, NEFA, and liver indices, while enhancing antioxidant enzyme activity (GPx, SOD) and reducing oxidative stress (MDA)	↓	Curcumin supplementation was safe and well-tolerated over 12 months, with no adverse effects, no hypoglycemia, and no significant changes in liver or kidney function	Yaikwawong et al. (2025)
IL6 and IL1β	Preclinical	50 mg/kg/24 rats	4 weeks	Curcumin treatment reduced liver enzymes (alanine aminotransferase, aspartate aminotransferase), improved oxidative status (lower malondialdehyde, higher glutathione peroxidase), and improved lipid profile (lower total cholesterol, triacylglycerides, low-density lipoproteins; higher high-density lipoproteins)	↓	No side effects were reported in this study *via* curcumin supplementation or treatment	Ghandour et al. (2025)
IL-13, IL-2, IL-17A, Fractalkine, TNF-α, IL-1β, IL-6, MCP-1	Preclinical	75 ± 10 mg/kg/not mentioned	12 weeks	Curcumin improved NAFLD by reducing inflammation, fibrosis, and NAFLD activity scores, while lowering AST. The reduction of RANTES—a key driver of NASH—strongly supports an anti-inflammatory mechanism	IL-13, IL-2, IL-17A, Fractalkine, ↑, while TNF-α, IL-1β, IL-6, MCP-1 ↔	Curcumin supplementation was well-tolerated with no adverse side effects. Body weight, food intake, and liver mass did not differ significantly between treatment and control groups. Although one rat died unexpectedly and three were euthanized due to weight loss or tumor, these events were not attributed to curcumin. Most other pro-inflammatory cytokines remained unchanged between the curcumin and control groups	Pickich et al. (2019)

Accumulating evidence indicates that curcumin alleviates NAFLD/NASH by suppressing chronic inflammation through multiple mechanisms. Its core action involves downregulating pro-inflammatory cytokines (TNF-α, IL-6, IL-1β) *via* inhibition of NF-κB, MAPK, and TLR signaling. Recent studies have expanded this understanding, revealing that curcumin also operates through a gut-hormone axis by suppressing GIP release from hypoxic intestinal tissue to reduce adipose inflammation. Additionally, curcumin exerts dual modulation of hepatocellular death pathways by promoting autophagy while inhibiting pyroptosis, thereby breaking the cycle of hepatocyte injury and cytokine-driven inflammation. These actions improve lipid metabolism, insulin sensitivity, and histopathological features of steatosis and fibrosis. Future research should focus on large-scale trials with bioavailable formulations and synergistic combinations to translate these mechanisms into effective therapies for advanced NASH.

## 4 Nicotinamide mononucleotide (NMN) and the modulation of immune-metabolic pathways

Recent advances have highlighted the role of NAD + precursors, such as nicotinamide mononucleotide (NMN), as potent modulators of the immune-metabolic landscape in obesity-related liver disease. While not a classical vitamin, NMN functions as a key intermediate in NAD + biosynthesis, influencing the activity of sirtuins (SIRT1), which are master regulators of inflammation and metabolism. Majeed et al. (2025) used an inducible SIRT1 knockout mouse model to dissect the pathways by which oral NMN supplementation mitigates diet-induced obesity and dyslipidemia, conditions intimately linked to NAFLD pathogenesis. Their comprehensive plasma proteomics analysis revealed that NMN’s effects are mediated through both SIRT1-dependent and independent mechanisms. Notably, NMN supplementation significantly altered the expression of multiple inflammatory mediators. Their causal analysis identified that NMN was linked to the upregulation of TH17-derived cytokines, including IL-17a and IL-17f, which were associated with the activation of master regulators such as FBXW7 and Sirt3. Concurrently, NMN supplementation led to the downregulation of several chemokines and inflammatory mediators, including Ppbp (CXCL7), Pf4 (CXCL4), Angpt1, and Thbs1, indicating a broad suppression of pro-inflammatory signaling. Furthermore, pathway enrichment analysis demonstrated that NMN significantly reverses obesity-induced alterations in immune-related gene networks (Majeed et al., 2025). This work provides direct evidence that NMN supplementation can modulate specific cytokine and chemokine pathways alongside metabolic pathways, offering a new framework for understanding how NAD + precursors influence the inflammatory milieu in NAFLD/MASLD through both SIRT1-dependent and independent mechanisms.

## 5 Clinical evidence for nutraceutical-mediated cytokine modulation in NAFLD/MASLD

The translational potential of cytokine-targeted nutritional strategies is increasingly supported by evidence from randomized controlled trials in patients with NAFLD/MASLD. Among n-3 PUFAs, DHA has demonstrated significant histological improvement in pediatric NAFLD, with Nobili and colleagues (Nobili et al., 2014) reporting reduced NAFLD Activity Score, steatosis, and ballooning after 18 months of supplementation, accompanied by decreased hepatic progenitor cell activation and pro-inflammatory macrophages through GPR120-mediated NF-κB inhibition, with significant reductions in serum TNF-α, IL-6, and IL-1β. Similarly, Kobyliak et al. (2018) found that an 8-week course of a multi-strain probiotic combined with omega-3 fatty acids significantly reduced the Fatty Liver Index, serum triglycerides, total cholesterol, and pro-inflammatory cytokines (TNF-α, IL-6, IL-1β, IL-8, and IFN-γ) in adults with T2D and NAFLD, with only minor, self-resolving adverse events reported. However, these positive findings were not replicated in a larger adult population by Alkhouri et al. (2024), where neither DHA alone (1.89 g/day) nor in combination with VE (1,000 mg/day) reduced hepatic fat fraction measured by MRI-PDFF over 6 months, highlighting the variability in treatment response across different populations and disease stages, although mild to moderate gastrointestinal symptoms were the most common adverse events across all treatment groups. The combination of fish oil and VD has shown encouraging results, with Guo and colleagues (Guo et al., 2022) demonstrating that 3 months of supplementation with fish oil (providing 2.34 g/day EPA + DHA) plus 1680 IU VD_3_ significantly reduced serum ALT, triglycerides, IR, and inflammatory markers including IL-1β and TNF-α compared to placebo, with no reported side effects, while the combination therapy showed additional benefits over fish oil alone for insulin sensitivity and inflammation. Qin and others (Qin et al., 2015) conducted a double-blind trial in 80 Chinese patients with NAFLD and hyperlipidemia, showing that 4 g/day of fish oil (containing 728 mg EPA and 516 mg DHA) for 3 months significantly reduced serum total cholesterol, triglycerides, apolipoprotein B, glucose, ALT, and GGT, while increasing adiponectin and reducing TNF-α, leukotrienes B4, fibroblast growth factor 21, cytokeratin 18 fragment M30, and prostaglandin E2. No serious adverse events were reported, although the corn oil control group showed a significant increase in serum creatinine. Importantly, the improvements in lipids, glucose, and liver enzymes were positively correlated with reductions in FGF21 and prostaglandin E2, suggesting these mediators may serve as biomarkers of therapeutic response.

VD supplementation has been evaluated in several randomized controlled trials. Sharifi et al. (2014) investigated high-dose VD3 supplementation (50,000 IU every 14 days for 4 months) in 53 adults with NAFLD, finding significant improvements in serum malondialdehyde and a trend toward reduced high-sensitivity C-reactive protein, but no significant effects on liver enzymes, IR, or steatosis grade on ultrasound, with no adverse effects reported. In a separate randomized controlled trial from India, Sakpal and others (Sakpal et al., 2017) evaluated VD supplementation in 81 patients with NAFLD and VD deficiency, finding that a single intramuscular injection of 60,000 IU VD combined with lifestyle modifications over 6 months significantly improved serum ALT levels and increased adiponectin compared to lifestyle modifications alone, while preventing the worsening of IR observed in the control group. Curcumin has emerged as one of the most extensively studied nutraceuticals for NAFLD, with consistent evidence of cytokine modulation. Yaikwawong and colleagues (Yaikwawong et al., 2025) reported in a 12-month randomized controlled trial in 78 patients with T2D and MASLD that 1,500 mg/day curcumin significantly reduced TNF-α, IL-1β, IL-6, and malondialdehyde levels at all follow-up intervals (3, 6, 9, and 12 months) while increasing antioxidant enzyme activities (glutathione peroxidase, superoxide dismutase) and significantly improving hepatic steatosis and liver stiffness measured by FibroScan. The supplementation was safe and well-tolerated over 12 months, with no adverse effects, hypoglycemia, or significant changes in liver or kidney function.

VE has been validated in diverse populations, with recent evidence supporting the use of lower doses. Song et al. (2025) conducted a multicenter, randomized, double-blind, placebo-controlled trial in 124 non-diabetic Chinese patients with biopsy-proven MASH, demonstrating that 300 mg of VE daily for 96 weeks significantly improved liver histology compared to placebo, with 29.3% of treated patients achieving the primary endpoint of histological improvement *versus* 14.1% in the control group. Notably, significant improvements were observed in steatosis, lobular inflammation, and even fibrosis stages, along with reductions in liver stiffness measurement and IL-6 levels, although TNF-α showed only a non-significant downward trend. The study reported 11 adverse events (seven in the VE group, four in the placebo) and 12 serious events, but none were treatment-related, and no cardiovascular events, stroke, or prostate cancer occurred. The trial design, as detailed by Zang et al. (2018), incorporated haptoglobin genotyping based on preliminary data showing that 65.71% of Chinese NAFLD patients carry the Hp 2–2 genotype—substantially higher than the 36% observed in Western populations—suggesting that Chinese patients may derive enhanced benefit from VE therapy due to this genetic predisposition.

These findings collectively underscore that while individual nutraceuticals demonstrate variable efficacy, the most pronounced benefits are observed in early-stage disease, in genetically predisposed populations, and when interventions are initiated before the establishment of advanced fibrosis. The safety profiles across these trials have been generally favorable, with most adverse events being mild, transient, and gastrointestinal in nature. However, the dissociation between anti-inflammatory effects and histological improvement observed in some studies, the variable response to EPA *versus* DHA, and the influence of genetic factors such as haptoglobin genotype on VE responsiveness highlight the critical need for personalized, biomarker-stratified approaches in future clinical trial design. These clinical observations provide strong translational validation for the cytokine-centric mechanistic framework presented in this review and support the potential of targeted nutritional strategies as effective adjuncts to first-line lifestyle therapies in NAFLD/MASLD management.

## 6 Safety considerations and translational limitations of nutraceutical therapy in NAFLD

Although nutraceuticals are widely regarded as safe due to their natural origin, the evidence summarized in this review indicates that their biological effects in NAFLD are highly context-dependent and not uniformly beneficial. Several experimental studies demonstrate that anti-inflammatory activity does not always translate into metabolic or histological improvement. For example, n-3 PUFA supplementation exacerbated hepatic inflammation in an MCD-induced NASH model, with increased intrahepatic TNF-α and MCP-1 expression and enhanced inflammatory cell infiltration (Provenzano et al., 2014). Similarly, Du et al. (2013) reported that although EPA reduced systemic TNF-α and IL-6 levels, it aggravated hepatic triglyceride accumulation and liver injury in the setting of impaired mitochondrial β-oxidation, highlighting a dissociation between cytokine modulation and lipid metabolism.

Variability in cytokine responsiveness has also been observed in vitamin-based interventions. While several studies demonstrated reductions in TNF-α, IL-6, and fibrotic mediators following VD or VE supplementation, others reported modest or statistically non-significant changes in key inflammatory markers (Fujimoto et al., 2008; Sakpal et al., 2017; Ma et al., 2019; Zakaria et al., 2025). These inconsistencies suggest that baseline deficiency status, disease stage, mitochondrial integrity, and metabolic phenotype may critically influence therapeutic responsiveness.

Moreover, differences in experimental design—including dietary models (HFD *versus* MCD), dosage regimens, intervention duration, and animal strain—may partially explain the divergent outcomes observed across studies. In some settings, improvements in oxidative stress markers were not accompanied by proportional reductions in inflammatory cytokines, indicating that antioxidant effects may not fully account for immunometabolic modulation (Fujimoto et al., 2008; Zakaria et al., 2025).

Collectively, these findings emphasize that nutraceutical therapy in NAFLD should not be considered universally benign or uniformly effective. Instead, patient stratification, biomarker-guided dosing, and careful metabolic phenotyping are likely required to optimize therapeutic benefit while minimizing potential adverse or paradoxical effects. Future well-designed clinical trials with standardized dosing and mechanistic endpoints are essential to clarify long-term safety and translational applicability.

## 7 Unresolved mechanistic controversies and knowledge gaps

Despite growing evidence supporting nutraceutical-mediated cytokine modulation in NAFLD/MASLD, several mechanistic uncertainties remain unresolved. A recurring observation across experimental and clinical studies is the dissociation between cytokine suppression and histological improvement. Significant reductions in TNF-α, IL-6, or IL-1β do not consistently translate into parallel improvements in steatosis or fibrosis, while some interventions ameliorate hepatic lipid accumulation without measurable cytokine normalization. This discrepancy challenges the assumption that anti-inflammatory activity alone is sufficient to reverse disease progression and suggests that metabolic and inflammatory pathways likely require simultaneous targeting.

Additional complexity arises from context-dependent and sometimes paradoxical responses. Although both EPA and DHA belong to the n-3 PUFA family, they demonstrate divergent mechanistic profiles: DHA consistently suppresses TGF-β/SMAD signaling and inflammasome activation, whereas EPA exhibits variable effects and may exacerbate steatosis under conditions of impaired mitochondrial β-oxidation. Similarly, for compounds such as VE and certain B vitamins, it remains unclear whether cytokine reductions result from direct immunomodulatory actions or occur secondarily through attenuation of oxidative stress. Clarifying whether antioxidant activity is upstream of cytokine suppression is critical for refining mechanistic interpretation and therapeutic positioning.

Moreover, most clinical trials continue to rely on liver enzymes and imaging endpoints without stratifying patients according to inflammatory phenotype or cytokine signatures. The absence of cytokine-guided trial design limits the identification of responder subgroups and obscures the mechanistic interpretation of outcomes. Collectively, these unresolved issues underscore the need for a structured and context-aware framework. Rather than merely summarizing nutraceutical interventions, the present review reorganizes evidence within defined cytokine-signaling axes—including NF-κB, NLRP3 inflammasome, TGF-β/SMAD, JAK/STAT, macrophage polarization, and ferroptosis pathways—thereby providing a mechanistic roadmap that extends beyond nutrient-class or outcome-based summaries and supports the development of cytokine-guided precision nutrition strategies in NAFLD/MASLD.

## 8 Conclusion

This review provides a comprehensive synthesis of current evidence supporting targeted nutritional interventions as a strategic approach for managing NAFLD/MASLD through modulation of cytokine-driven pathways. The nutraceuticals discussed—n-3 fatty acids (EPA and DHA), vitamins D, E, and B, and curcumin—demonstrate the capacity to rebalance the dysregulated hepatic immune environment. Their therapeutic potential is primarily rooted in suppression of key pro-inflammatory mediators, including TNF-α, IL-6, and IL-1β, through inhibition of central signaling hubs such as NF-κB, MAPK, and the NLRP3 inflammasome.

Beyond anti-inflammatory effects, these compounds exert multi-dimensional benefits: enhancement of antioxidant defenses (vitamin E and D, curcumin), promotion of anti-inflammatory macrophage polarization (VD, DHA), direct inhibition of hepatic stellate cell activation and fibrogenesis (DHA, VD, curcumin), and improvement of metabolic parameters, including insulin sensitivity. Collectively, these mechanisms translate into improvements in steatosis, lobular inflammation, and fibrosis in both experimental models and selected clinical trials, supporting their role as adjuncts to first-line lifestyle interventions.

However, a central conclusion of this review is that the therapeutic efficacy of nutraceuticals is not universal but highly context-dependent. Outcomes are influenced by compound specificity and formulation (e.g., purified EPA-ethyl ester *versus* mixed fish oil), dosage, treatment duration, genetic background (such as Hp 2-2 genotype–dependent responses to VE), and, critically, the patient’s underlying metabolic status. The paradoxical effects of EPA exemplify this complexity: while anti-inflammatory and antifibrotic under metabolically intact conditions, EPA may exacerbate hepatic lipid accumulation when mitochondrial β-oxidation is impaired.

Emerging evidence further expands this mechanistic landscape. Curcumin appears to modulate novel gut–liver endocrine axes by suppressing GIP release from hypoxic intestinal tissue, thereby attenuating adipose inflammation, and by coordinating hepatocyte cell death pathways—promoting autophagy (↑BECN1, ↓p62) while inhibiting pyroptosis (↓Gasdermin D, ↓caspase-1, ↓TNF-α, ↓IL-1β). In parallel, NAD^+^ precursors such as nicotinamide mononucleotide (NMN) demonstrate the ability to reprogram immune–metabolic signaling through both SIRT1-dependent and independent mechanisms, including modulation of TH17-derived cytokines (IL-17A, IL-17F) and chemokines such as CXCL7.

By organizing current evidence within a cytokine-centered mechanistic framework and critically addressing context-dependent and sometimes paradoxical responses, this review moves beyond descriptive summaries of nutraceutical efficacy. Instead, it highlights unresolved mechanistic questions and underscores the need for cytokine-stratified, precision-based nutritional strategies to optimize NAFLD/MASLD management. Future research should prioritize well-designed, stage-specific clinical trials incorporating histological endpoints, metabolic phenotyping, and immunological stratification to translate these mechanistic insights into personalized therapeutic protocols.

## 9 Future perspectives

Future research must urgently bridge the translational gap between compelling preclinical mechanistic evidence and consistent, clinically meaningful outcomes. The foremost priority is the design of large-scale, long-term randomized controlled trials incorporating histological endpoints as primary outcomes. Demonstrating improvements in lobular inflammation, hepatocellular ballooning, and fibrosis—the defining features of NASH progression—will be essential to establish true therapeutic efficacy, rather than relying solely on serum biomarkers or liver enzyme reductions, which remain imperfect surrogates of histological benefit.

A critical direction for future investigation involves defining the metabolic, immunological, and genetic determinants of treatment responsiveness. Identifying predictive biomarkers—including genetic variants (e.g., Hp genotype), markers of mitochondrial function, inflammatory cytokine profiles, and integrated metabolic signatures—will enable stratification of patients according to likely benefit. Such an approach is fundamental to advancing precision nutrition strategies, ensuring that specific nutraceuticals are administered to the appropriate patient population and disease stage.

In parallel, formulation science must be prioritized to overcome persistent bioavailability limitations, particularly for compounds such as curcumin and certain lipid-based interventions. Future randomized controlled trials (RCTs) should incorporate bio-optimized delivery systems (e.g., phospholipid-complexed DHA, curcumin combined with absorption enhancers such as piperine, or nanoemulsified VD preparations) that enhance tissue targeting and pharmacodynamic activity. Standardizing formulation, dosing, and treatment duration will be essential to reduce heterogeneity across clinical studies and to enable reproducible therapeutic outcomes in NAFLD/MASLD.

Collectively, these efforts will shift the field from empirical supplementation toward mechanism-guided, cytokine-stratified nutritional therapeutics capable of integrating metabolic and immune profiling into clinical decision-making.
